# An Atomistic Study of Reactivity in Solid-State Electrolyte Interphase Formation for Li/Li_7_P_3_S_11_

**DOI:** 10.1021/acs.jpcc.5c03589

**Published:** 2025-09-03

**Authors:** Bryant Y. Li, Vir Karan, Aaron D. Kaplan, Mingjian Wen, Kristin A. Persson

**Affiliations:** † Department of Materials Science and Engineering, 1438 University of California Berkeley, Berkeley, California 94720, United States; ‡ Materials Science Division, 1666 Lawrence Berkeley National Laboratory, Berkeley, California 94720, United States

## Abstract

Lithium metal batteries offer superior volumetric and gravimetric specific capacities compared to those based on traditional graphite anodes. Although advancements in solid-state electrolytes address safety concerns, challenges remain, particularly regarding interphase formation in lithium metal anodes. This work presents a computational framework based on high-throughput first-principles density functional theory and machine-learning interatomic potentials (MLIPs) including automated iterative, active learning to enable robust computational exploration of interphase formation between lithium metal anodes and an inorganic solid-state electrolyte. As a demonstration, we apply the framework to a Li/Li_7_P_3_S_11_ interface and find that it accurately identifies the experimentally observed, thermodynamically stable interphase products as well as their overall spatial arrangement within a heterogeneous, amorphous layered structure, with Li_2_S domains of nanocrystallinity. Our simulations show two stages, a fast and slow diffusion reaction regime, that corroborate the relative phase formation rate of Li_*x*_P, Li_2_S, and Li_3_P. Using the Onsager transport theory, we capture time-dependent ionic diffusion within the reacting interface, including cross-correlation effects. We found that cross-correlation effects between Li–P and P–S ionic motion significantly influence P-ion diffusion, making it highly sensitive to the local environment and potentially leading to “kinetic trapping” of Li–P phases. The passivation of the interface is shown as the ionic fluxes all approach zero, effectively halting interphase growth.

## Graphical abstract

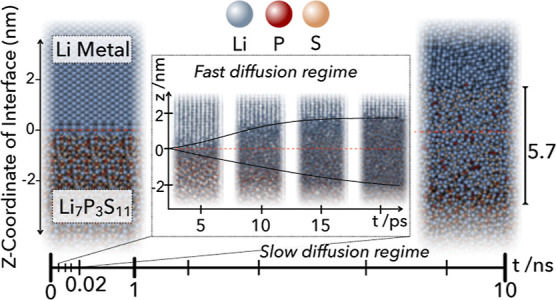

## 1 Introduction

As the global demand for energy and energy storage increases, developing high-capacity technologies needs to be accelerated, e.g., through fundamental understanding and predictive modeling techniques. All-solid-state batteries (SSBs) with lithium (Li) metal anodes have emerged as leading candidates for high-capacity, rechargeable energy storage systems^1^ due to their projected improvement in volumetric and gravimetric energy densities.^2–5^ Li metal SSBs benefit from the low electrochemical potential (−3.04 V vs SHE) and high theoretical specific capacity (3860 mAh g^–1^)^6^ of the Li metal anode, in addition to improved safety as compared to conventional Li-ion batteries that rely on flammable liquid electrolytes.^7–9^

Progress has been made in the design and discovery of novel solid-state electrolytes (SSE)^10–14^ via prediction of their ionic conductivity,^15^ thermodynamic stability,^16,17^ and their in operando, ex situ characterization^18–22^ and modeling^23–25^ of interfacial evolution. It has been shown that SSBs face challenges from the reactivity of the SSE–anode interface, leading to the formation of interfacial products with inferior ionic transport and subsequently decreasing rate capability and capacity.^26,27^ This issue highlights the importance of modeling the initial stages of solid-state reactivity at the metal anode–SSE interface to better understand the interface stability and optimize the performance of current and future SSBs.

First-principles methods, such as density functional theory (DFT),^28,29^ offer accurate predictions of thermodynamic properties, reactivity, and electronic structure but are constrained by high computational costs that scale unfavorably with system size.^30^ For example, ab initio molecular dynamics (AIMD) is currently limited to picosecond time scales for periodic systems with hundreds of atoms, particularly in simulating interfacial reactivity.^23,24,31^ On the other hand, machine-learning interatomic potentials (MLIP) have recently demonstrated promise when trained on DFT data, such as AIMD trajectories, and can extend into classical MD time and length scales.^32–34^ Importantly, since such ML methods leverage DFT data to describe the shape of the DFT potential energy surface (PES), it is possible to investigate dynamic properties such as reactivity and diffusivity from first principles.^35–39^ Researchers have developed various MLIP models with distinct architectures to accurately capture local bonding interactions and predict the energies of diverse material systems.^32,40–42^

In this work, we present an automated, computational framework that leverages the atomic cluster expansion (ACE)^43–45^ MLIP architecture. This framework incorporates ACE’s inherited active learning infrastructure to systematically generate an interfacial reactivity data set intended for extended time- and length-scale simulations, suitable for solid-state room-temperature reactions relevant for SSBs. The framework also accounts for crystalline and noncrystalline structures along the chemical space of the interface, ensuring comprehensive data set coverage.^46,47^ We apply the framework to gain a fundamental understanding of atomic mechanisms that influence the interfacial chemistry and morphology of the Li/Li_7_P_3_S_11_ solid-state battery system, simulating the interfacial reaction evolution and quantifying the products formed at the interphase. We find that the reaction between Li and Li_7_P_3_S_11_ passivates through the formation of a heterogeneous, layered, amorphous interphase with dominant phases emerging in the sequence: Li/Li_3_P–Li_*x*_P/Li_2_S/mixed Li_2_S–Li_*x*_P/Li_7_P_3_S_11_. The resulting interphase morphology exhibits regions containing higher concentrations of phases such as Li_2_S and Li_3_P, with the spatial distributions that align with available in situ X-ray photospectroscopy (XPS) experimental observations.^19,48^ Notably, while the SEI predicted by our model is chemically similar to experimental observations, it does not exhibit the full crystallization of Li_2_S as observed in other models^38,49^ but rather nanocrystalline domains of Li_2_S embedded in an amorphous matrix of the same composition. We further obtain ionic fluxes from the simulations of amorphous interface constituents and derive Onsager transport coefficients. Based on this analysis, we suggest that the spatial distribution of interfacial P species results in part from the correlative ionic transport between Li, S, and P ions which effectively presents “kinetic traps” for P ions. Overall, our framework lays the groundwork for enabling long-time-scale, accurate “digital twins” for room-temperature solid-state reactivity, uncovering the mechanistic evolution of SEI formation.

## 2 Methodology

### 2.1 Generalized Workflow for Interfacial Reactivity Data Sets

In this work, we propose a novel generalized framework for generating DFT data sets, designed to train MLIPs with the use of active learning, to simulate solid-state interfacial reactivity. The framework leverages the computational infrastructure provided by the Materials Project,^50^ which allows for the production of large batches of accurate DFT calculations to sample the potential energy landscape across the interfacial chemical system. The generation of high-throughput DFT data sets, both static and dynamic calculations, was performed using purpose-built Python packages, such as pymatgen,^51^ jobflow,^52^ atomate2,^53^ fireworks,^54^ and custom code (see sections “DFT Computational Details” and “Classical Molecular Dynamics” for specific methods used for DFT calculations and MD simulations). The choice of the MLIP architecture can be substituted if desired. The three main components of the framework (configurational sampling, model training, and applications) are shown in Figure 1.

**Figure 1:**
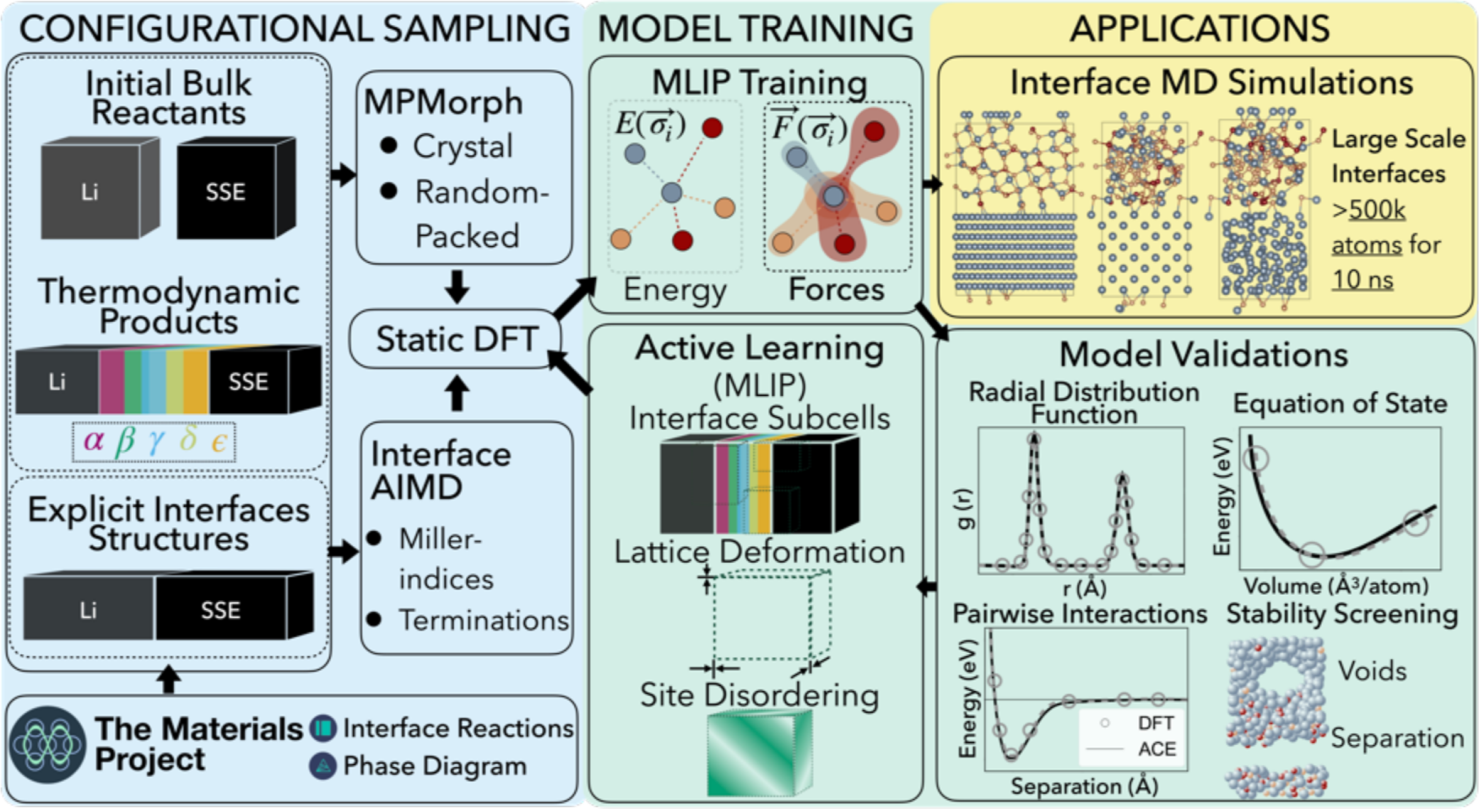
Illustration of the framework used to design an MLIP to model interfacial reactivity which includes (1) initial configurational sampling: user-specified samples of equilibrium and nonequilibrium structural configurations based off of the reactant compositional space and thermodynamically predict phase presence, (2) model training: iterative MLIP training with an active learning schema and validation from RDFs, EOS, pairwise interactions, and stability screening, and (3) applications: direct simulations of interfacial structures in MD at extended length and time scales.

The framework workflow begins with configurational sampling as the first stage: the initial bulk reactants of the interfacial reactivity system are selected and then employed to identify thermodynamically favorable chemical reactions and phases across the system’s compositional space. We benefit from the Materials Project’s Interface Reaction^16,50^ and Phase Diagram^55^ applications, which enable us to generate a list of thermodynamically possible product phases that are able to form from the bulk reactants, shown in Figure 2. These phases are obtained in both crystalline and amorphous forms using MPMorph.^56,57^ In addition to the bulk phases, we also create explicit interfacial structures via the CoherentInterfaceBuilder class in pymatgen, distinctively generated via Miller indices and termination matches that obey symmetry.^58,59^ The atomic configurations of these structures are subsequently perturbed using AIMD simulations to sample nonequilibrium states, capturing critical structure–property relationships essential for informing kinetic effects for MLIP models. The Li–Li_7_P_3_S_11_ specific data set is explained in Section 2.2 “Li/Li_7_P_3_S_11_ Ab Initio Training Data Set.”

**Figure 2:**
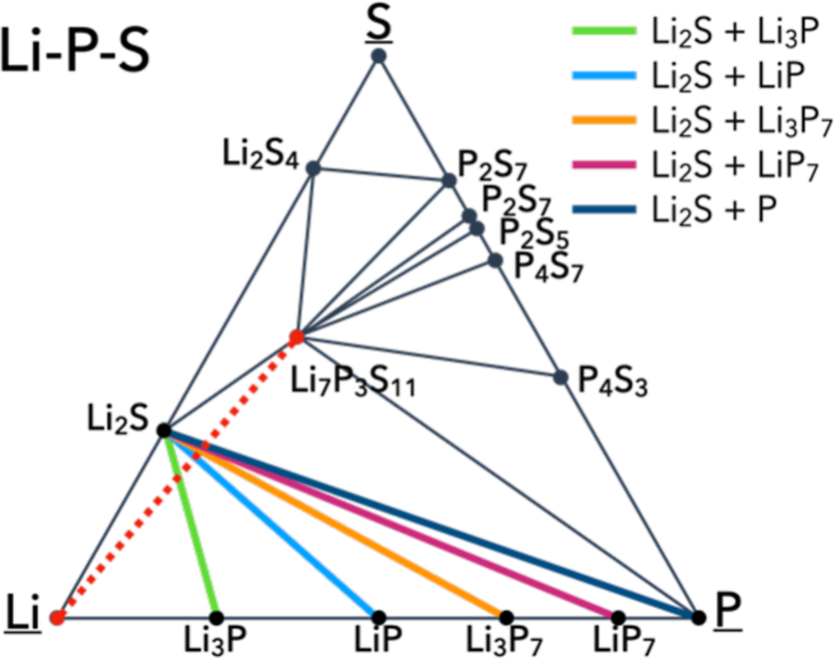
Phase diagram of the Li–P–S ternary phase space based off of data available from the Materials Project.^50^ The red dashed line indicates the initial tie-line constructed between the selected reactants of Li and Li_7_P_3_S_11_. Other color lines indicate chemical reactions that were favored thermodynamically and their respective product phases.

The second stage of the workflow involves model training: the data set from the configurational sampling stage is processed to train an MLIP using the ACE architecture. To improve accuracy in reactivity mechanics and kinetics, the training procedure emphasizes errors in interatomic forces. The model is accepted only after achieving the required convergence for energy and force, measured by root-mean-squared error (RMSE) and mean absolute error (MAE) against a test data set.^32,40–42,60^ We then validate the model with several metrics: pairwise interactions, radial distribution functions (RDF), equations of state (EOS), and interface stability screenings. All pairwise interactions were plotted against DFT data to reflect the model’s ability to capture the PES. The RDFs are obtained for both the crystalline and amorphous phases to gauge the accuracy of the interatomic potential’s ability to map bulk phases. The EOS benchmarks the energy, bulk moduli, and pressure predictions. Finally, the model is screened for stability during MD simulations of interface structures by measuring the localized errors across extended pressure and volume ranges, whereby errors such as void formation and phase separation are indicative of MLIP failure modes. The four aforementioned metrics validate the physicality properties of the MLIP’s dynamic performance and its predictive capabilities of interphase formation. After validation, we implemented an active learning screening detailed in the section “Active Learning”, where we added the data to retrain the next-generation model. We found that this iterative scheme allows for robust simulations of the interfacial reactivity over time.

The third stage of the workflow involves extended time-scale MD simulations to extract interfacial reactivity and kinetics. The primary objective is to perform an MD simulation on a Li/Li_7_P_3_S_11_ interface structure that can be extended into the nanosecond time-scale regime for over 500,000 atoms. These structures can include various degrees of crystallinity given the data set the MLIP is trained on. Kinetic information, such as diffusion coefficients and Onsager transport coefficients, can be obtained through both small- and large-scale simulations provided the simulation time scale captures sufficient ionic mobility statistics.

### 2.2 Li/Li_7_P_3_S_11_ Ab Initio Training Data Set

Including both the initial and subsequent active learning steps, a total of 11,596 structures were generated to describe the Li–P–S compositional space. An initial set of 2,030 structures was first generated and calculated via DFT to train the first-generation Li–P–S potential. This initial set of structures was constructed via the configurational sampling scheme in Figure 1, by selecting BCC Li and Li_7_P_3_S_11_ as the initial reactant phases to reflect experimental observations.^18,48,61–64^ The phases Li_2_S, Li_3_P, LiP, Li_3_P_7_, LiP_7_, and Li_3_PS_4_ were identified as plausible reaction products via the workflow which aligns with experimental in situ XPS observations.^18,19^ The reactant and product phases were structurally optimized in DFT, then simulated at 300, 900, and 1500 K in AIMD for 20 ps, and subsequently resampled every fiftieth frame to be recalculated in DFT with more strict computational parameters (see the “DFT Computational Details” section). The MPMorph workflow^56,57^ was also applied to the aforementioned reactant and product phases to generate amorphous structures and systematically recomputed with static DFT at every fiftieth frame. Interfaced Li/Li_7_P_3_S_11_ structures were generated via the CoherentInterfaceBuilder from pymatgen for the lowest energy planes with maximal surface area, which were the (100), (101̅), and (010) planes for Li_7_P_3_S_11_ ^62^ and the (100) and (110) planes for BCC Li.^65^ We enumerated all unique slab termination sites. All structures were constructed in a “sandwich” configuration with a 1.5 Å spacing between two bulk regions with an orthogonal unit cell of size ∼100–400 atoms. The remaining data were systematically generated with interfaced, disordered, and rescaled structures of these phases via the active learning framework (see “Active Learning” section).

### 2.3 DFT Computational Details

This section details the DFT computational tools used in generating structures in the configurational sampling stage of the workflow. All DFT calculations and AIMD simulations were performed using the Vienna ab initio simulation package (VASP).^66–69^ Crystalline structures were first queried via the Materials Project^50^ and modified and processed with the pymatgen^51^ Python package. All structures were calculated with VASP workflows provided by atomate2 ^53^ and managed by Fireworks^54^ for high-throughput performance. The data has numerical parameters compatible with the Materials Project^50^ (v2023.11.1 database release). The Perdew–Burke–Ernzerhof generalized gradient approximation (GGA) for solids (PBEsol)^70^ was used for all calculations, with a plane-wave energy cutoff of 680 eV, a *k*-point density of 64 per Å^–3^, and an electronic self-consistency convergence criterion of 1 × 10^–5^ eV. An ionic relaxation convergence criterion of 2 × 10^–2^ eV/Å in the forces was used for all structural optimizations. The static DFT electronic structure calculations and structural optimizations use the StaticMaker and RelaxMaker from the atomate2 ^53^ package, inherited from the VASP module’s static set in pymatgen.^51^ For more details on the DFT calculations, we refer to Tables S2 and S3.

All AIMD simulations were performed non-spin-polarized, using a 2 fs time step with the Nosé–Hoover thermostat in the *NVT* ensemble using the Γ-point only. Structural optimizations are conducted before AIMD simulations of crystalline systems. Crystalline supercell structures (∼100 atoms) were simulated at temperatures of 300, 900, and 1500 K. These temperatures access high-temperature regimes without melting the phases, ensuring a higher likelihood of accessing perturbed structural configurations to maximize MLIP data set sampling.^71^ Structures with significant disorder, such as amorphous structures, were simulated in AIMD via the MPMorph workflow.^56,57^

The MPMorph workflow has been designed and extensively benchmarked to systematically generate noncrystalline structures for DFT purposes.^56,57,72^ Random initial structures are generated at the given phase composition via PACKMOL,^73^ then isovolumetrically rescaled to perform a series of 4 ps *NVT* AIMD runs to fit the equation of state at the specified temperature. A predicted equilibrium volume is acquired via the Birch–Murnaghan equation-of-state fit. A 20 ps AIMD production run is simulated upon ensuring energy and density convergence by an iterative loop. For more details on AIMD calculations, we refer to the MDMaker and MPMorphMDSet class in atomate2.

### 2.4 Classical Molecular Dynamics

This section details using Atomic Simulation Environment (ASE)^74^ molecular dynamics (MD) and machine-learning-performant atomic cluster expansion (ML-PACE) for large-scale atomic/molecular massively parallel simulator (LAMMPs)^75^ and their input settings to start the simulation. Classical MD simulations were performed using ASE for smaller structures with plugs-in built by pacemaker^76–79^ and atomate2.^53^ MD simulations used LAMMPS for larger simulations, with plug-ins built from pacemaker.^76–79^

Preliminary tests of the fitted potential were conducted using pacemaker adapted with ASE, a nonparallelized version of the MD code. All ASE-based MD simulations were performed with the Langevin thermostat^80^ and a 1 fs time step. Custom code ASE PACE in atomate2 was also adapted to accelerate these simulation workflows. Simulations were consistently performed in the *NVT* ensemble for all of the ASE-MD simulations.

Extended time-scale MD simulations used the ML-PACE implementation in the LAMMPS simulation software.^75^ A Langevin thermostat^80^ was used for all MD simulations with the Li–P–S ACE MLIP, using a 1 fs time step unless otherwise noted. We simulate a system comprising 500,000 atoms, constructed via the CoherentInterfaceBuilder class in pymatgen, for a duration of up to 10 ns with the lowest energy surfaces (100/100) for Li/Li_7_P_3_S_11_, as this interfacial contact is the most likely case observed in experimental studies.^18,19^ All simulation results and interfaced structures were rendered with Ovito^81^ 3.8.3.74. We do not recommend using the potential developed here with time steps greater than 1 fs/step. Unstable behavior was observed for simulations at 1500 K with 2 fs time steps, even with reasonable starting geometries.

### 2.5 Active Learning

The active learning step begins with the use of AIMD and MLIPs to sample disordered local configurations present in the bulk and in the interface. The integration of AIMD and MLIPs permits the flexible sampling of highly disordered states, significantly reducing the overall computational runtime. The first batch of actively learned configurations comes from simulations of the original bulk crystal structures at elevated temperatures, employing isovolumetrically rescaled lattices. The second batch comes from MPMorph MD simulations of accessible phases composition, which were also simulated under similar elevated temperatures. The configurations generated serve as a basis for exploring nonequilibrium configurations accessible for the bulk phases during solid-state reactivity. The third batch of configurations are sampled from MD simulations of interface structures of >100,000 atoms. Simulations of interface structures pose significant challenges due to their inherently large size, which can lead to computational constraints when utilizing electronic structure-based calculation methods. To address this, we divided each frame of the MD simulations into smaller subcells containing approximately 100 atoms each. Each subcell is padded by a smaller layer of vacuum (0.5 Å) to prevent overcoordination near the new periodic boundary conditions (PBC) of the subcell. These subcells are therefore populated with local environments present only in the interphase, aiming to faithfully represent the high disorder and dynamically reactive state. The final stage of the active learning component involves selecting relevant structures from the three batches of configurations mentioned before. We utilize the D-optimality criterion,^43^ as proposed by Lysogorskiy et al.,^82^ implemented in pacemaker^44,45^ to identify necessary structures from previous AIMD/MLIP sampling methods for recomputation in static DFT. The D-optimality criterion is based on the **B**-matrix, a reduced symmetrically invariant representation of spherical harmonic basis functions that captures the continuous interactions within a cluster of atoms (see “ACE Potential Architecture” for details). When a configuration is well-represented in a data set, the weights and coefficients for the **B**-matrix are well-tuned and refined; conversely, if these interactions are not well-captured by the data set, the coefficients for the basis functions are poorly defined and undertrained. The D-optimality criterion evaluates the certainty of the model describing new structural configurations based on the enclosed space defined by the trained **B**-matrix. The extrapolation grade, γ, measures how well-defined a configuration is in the model via the D-optimality criterion. Configurations where the D-optimality criterion evaluates an extrapolation grade of 1 < γ < 2.5 were chosen to propagate the next generation of training data. This range of γ was chosen to ensure that the structures found from the active learning loop fall outside the confidence interval of the current ACE model. The data are recalculated with the same atomate2 StaticMaker to ensure compatibility within the initial training data (see “DFT Computational Details”). This active learning loop is applied three times in this study; however, it could theoretically be performed for as many iterations as needed to develop a more precise MLIP.

### 2.6 Model Training and Parametrization

The training parameters of the ACE model in this work include 350 basis functions per element, a cutoff radius of 6.0 Å, and the Finnis–Sinclair-shifted-scaled embedding in pacemaker, using the exponential parameters implemented by Erhard et al.’s work.^83^ The model fitting was conducted in a hierarchical manner, with low-body order fits completed first and higher-body functions added and fitted iteratively. The model training is performed in two stages: an initial coarse model is obtained by placing κ = 99% weight on the force loss function; the second stage places 90% weight on the force loss function. As we are primarily concerned with modeling reactivity while retaining MD stability, we have chosen a much heavier force weighting than conventional MLIP models that target relative energetics.

After training, the model undergoes a set of benchmarks and validation to evaluate the model’s stability and accuracy against DFT data (see the “Model Validation” section). If the model fails, then the active learning stage is initiated, and new structures are generated and processed through pace select (a built-in function of pacemaker). This function uses the extrapolation grade to identify a set of relevant structures to augment the current set (see the “Active Learning” section). DFT single-point (static) calculations are then performed on these structures. The next iteration ACE potential is refit with the same parametrization listed above. This process continues until the final potential reaches the desired performance metrics, with convergence achieved when the loss function Λ reaches below the 0.2 threshold. The final potential, which comprises 1,634 basis functions, has its architecture detailed in Table S1.

### 2.7 Onsager Ionic Transport Analyses

To study ionic diffusion—including correlated ion transport during interfacial reactions—we compute the Onsager transport matrix proposed by Fong et al.^84,85^ and performed in the context of solid-state reactions by Karan et al.^86^ We performed MD simulations on amorphous structures, corresponding to the 20 stable crystalline polymorphs in the Li–P–S phase diagram from the Materials Project, with a target supercell size of 100 atoms. The Onsager transport coefficients can be calculated from MD trajectories using the differential form of the Green–Kubo relations:^84^

1 $$L_{ij}=\frac{1}{6k_{B}VT}\lim_{t\to \infty }\frac{d}{dt}\left\langle \sum_{\alpha }\left[r_{\alpha }^{i}\left(t\right)-r_{\alpha }^{i}\left(0\right)\right]\cdot \sum_{\beta }\left[r_{\beta }^{j}\left(t\right)-r_{\beta }^{j}\left(0\right)\right]\right\rangle$$

Here, *V*, *T*, and *k*_B_ are the volume, temperature of the system, and Boltzmann constant, respectively. **r**_α_^*i*^(*t*) – **r**_α_^*i*^(0) is the displacement of the α^th^ particle of specie *i* at time *t*. Diagonal terms of **L** (*L*_*ii*_) provide a measure of the net transport of ion *i*, while off-diagonal terms (*L*_*ij*_) measure the cross-ion transport effects, which lead to a coupling between the ionic fluxes of different species.

The motion of an ion across the interface can be quantified by its ionic flux, which in the framework of linear irreversible thermodynamics is given by

2 $$J_{i}=-\sum_{j}L_{ij}\nabla \tilde{\mu_{j}\;}$$

Here, **L** = {*L*_*ij*_} is the Onsager transport matrix and $\nabla \tilde{\mu_{j}\;}$ is the chemical driving force on ion *j* across the interface, which we approximate as the distance between Li and Li_7_P_3_S_11_ phases from the chemical potential diagram^87^ (refer to the Supporting Information for numerical values chemical potential gradients used). Accurate statistics for fitting Onsager coefficients typically require extended time-scale simulations and we refer to Karan et al.^86^ for more details.

## 3 Results and Discussion

### 3.1 Model Validation

A central goal of this work is to establish benchmarks for assessing the accuracy of MLIPs in modeling solid–solid reactivity. Model validation of MLIPs is currently standardized by RMSE and MAE values in energy and force prediction.^46,47,88^ The RMSE for the final generation ACE model is 119.5 and 269.8 meV/Å, for energy and forces, respectively.^18^ The MAE values are 81.5 meV/atom for energy and 166.6 meV/Å for forces. These metrics are gathered by a 90%–10% split of the training and test data prior to model training. Although these metrics are considered inadequate among large universal interatomic potential models and some small-scale interatomic potentials, the higher training error is attributed to the emphasis on reactivity prediction and nonequilibrium structures, a domain which previous models have not accessed. We refer to benchmark Figure S1 of the final ACE potential against DFT data for details.

While energy and force errors are suitable equilibrium metrics, practical use of MLIP dynamics requires a different set of near- and nonequilibrium metrics. We choose the EOS and RDF as metrics of near-equilibrium properties and the transport coefficients as a nonequilibrium metric. We include the pairwise interaction energy plot to validate the model smoothness and stability at compressed pairwise regimes. These are measurable properties for which the model is not trained. The benchmarks are carried out for Li_2_S, Li_3_P, LiP, Li_3_P_7_, LiP_7_, Li_7_P_3_S_11_, and Li.

For the pairwise interactions shown in Figure 3, we observe a smooth, continuous fit for the trained ACE potential, and it displays an overall good agreement with the DFT data for the reference system. The discrepancies between DFT and the ACE model for Li–S interactions likely stem from undersampling, as these interactions are present only in Li_2_S, Li_7_P_3_S_11_, and active learned interphase structures. Notably, the curvature of the pairwise interactions closely aligns with DFT calculations, whereas the differences in binding energy well depth are more pronounced—both likely resulting from the model’s training emphasis on forces. For the EOS in Figure 4, the model performs a satisfactory fit compared to the DFT counterpart, as evidenced from the matching EOS fit curvatures. This EOS fit also illustrates the extensive volumetric coverage within which the model performs similarly to DFT, showcasing its functionality across a wide range of volume and pressure variations. The latter is particularly important for a reactive solid-state interface, where the final products exhibit a significantly different volume than the original. This broad coverage reflects the model’s stability under dynamic configurations with compromises made to the precision of energy predictions. The pairwise interaction curves and EOS reveal model stability in the highly compressed limit. The RDFs also suggest robust crystalline stability for all the phases at 300 K, showing excellent agreement with the reference data. Figure 5a,b demonstrates that low-symmetry structures such as Li_3_P_7_ and Li_7_P_3_S_11_ show dynamical stability in agreement with DFT at finite temperatures. We performed the stability screening metric by first sampling all identifiable crystalline phases in the data set, acquired their amorphous counterpart, and also generated different sizes (1000–7000 atoms) of Li/Li_7_P_3_S_11_ interfaces with distinct termination planes. All phases listed above were screened in 1 ns *NVT* and *NpT* ML-MD runs to ensure no void formation and phase separation occurred.

**Figure 3:**
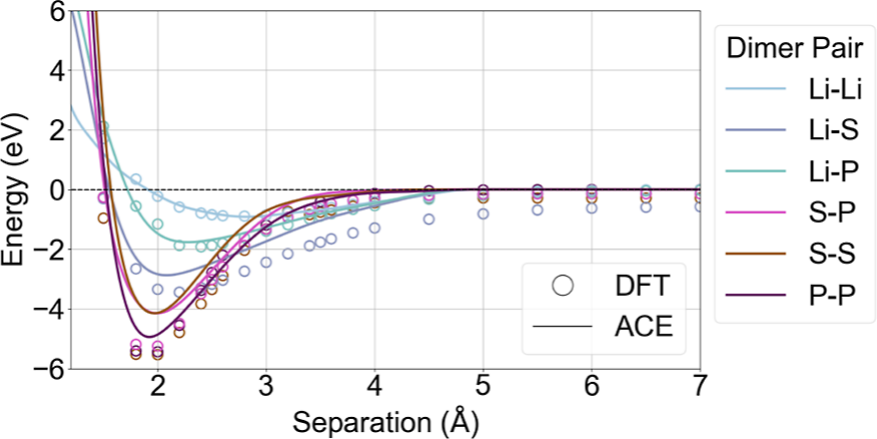
Binding energy curve illustrating pairwise interactions between all pairs in the Li–P–S system.

**Figure 4:**
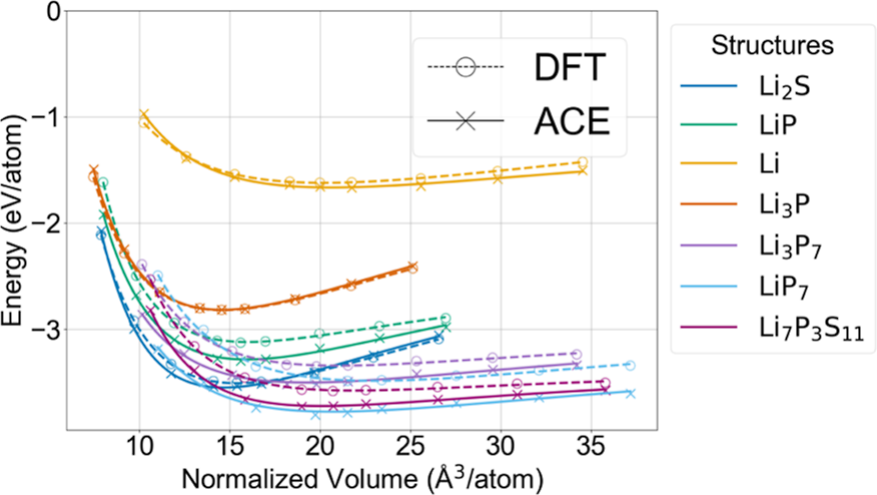
Equation of state for the known crystalline structures in the Li/Li_7_P_3_S_11_ chemical space, plotted with respect to normalized volumes.

**Figure 5:**
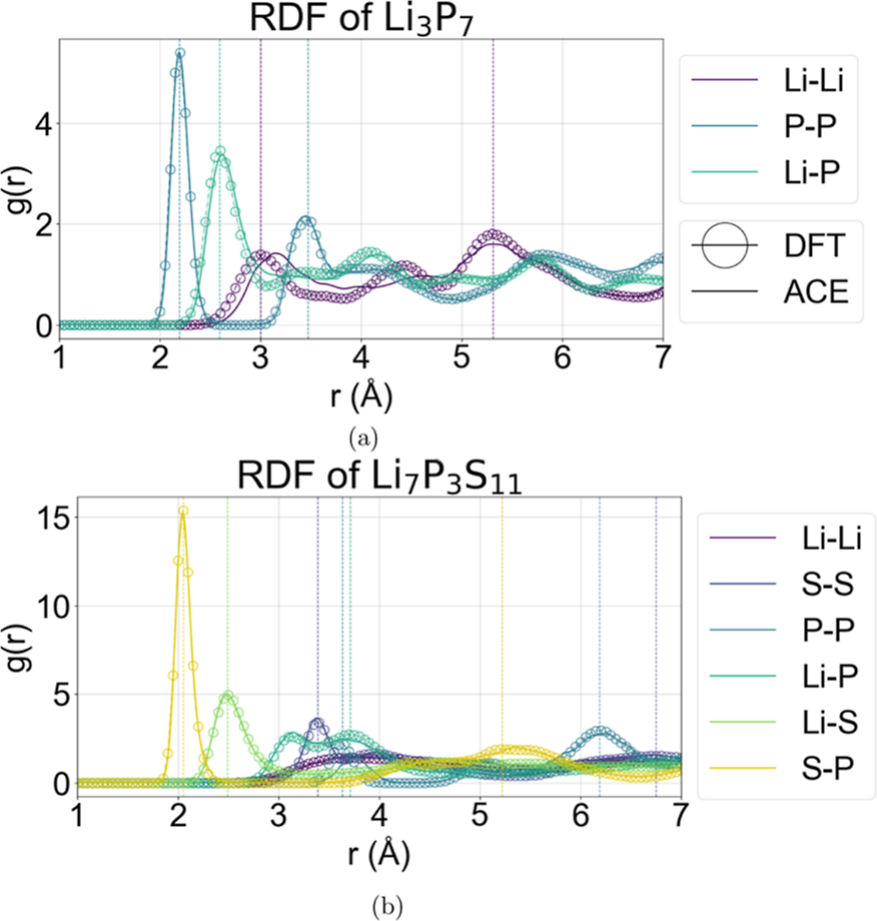
Radial distribution functions for (a) Li_3_P_7_ and (b) Li_7_P_3_S_11_ using the ACE MLIP and AIMD at 300 K, performed for 20 ps. See Figure S2 for the other Li–P–S phases.

### 3.2 Interphase Formation Simulation and Transport

For the extended time- and length-scale interphase formation simulation, we construct a unit cell with the lowest energy surfaces (100/100) for Li/Li_7_P_3_S_11_ with 500,000 atoms and performed a simulation at 300 K in the *NpT* ensemble (see Methodology: “Classical Molecular Dynamics” for details). As Figure 6 shows, the simulation initially presents a fast diffusion regime, followed by a slow diffusion regime. The fast diffusion regime completes within 20 ps and forms an approximately 6 nm thick barrier layer of the interphase. We find that the barrier layer grows into the Li metal and the Li_7_P_3_S_11_ SSE at different rates and that the growth rates change as a function of time. In the fast diffusion regime, the growth into the Li layer is slower than into the Li_7_P_3_S_11_ layer. This behavior is reversed as the process enters the slow diffusion regime, whereby the growth into Li occurs at a faster rate than that into the SSE. This difference in growth direction may be attributed to fast transport of Li in the SSE Li_7_P_3_S_11_. As the passivation layer builds, the Li diffusion within it slows down and growth increases into the Li layer.

**Figure 6:**
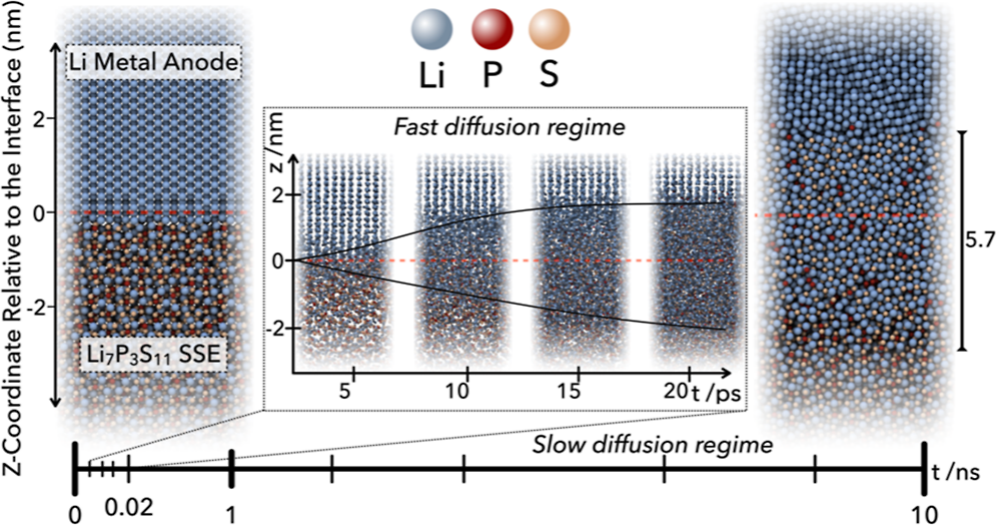
*NpT* simulation of the (100)/(100) Li/Li_7_P_3_S_11_ interface over 10 ns using molecular dynamics with the final generation of fitted machine-learning interatomic potentials (MLIP). The snapshots showcase the evolution of the simulation at specific timestamps. The fast diffusion regime, observed within the first 20 ps, indicates the initial formation of the interphase and the growth of the interphase across the original interface boundary. In contrast, the slow diffusion regime reflects the stabilization of the interphase region extending up to 10 ns.

Figure 7 presents the net ionic flux over the interface for the three elemental species during the 10 ns *NpT* simulation. This data evidences the fast and slow diffusion regimes observed within the simulation, whereby fast diffusion is observed as a sharply peaked flux of Li toward the Li_7_P_3_S_11_ SSE (negative direction), and P and S ions show a strong reverse flux toward the Li metal anode (positive direction). Both Li and S ions exhibit a significantly larger flux at the beginning of the interface reaction, compared to P ions. In the slow diffusion regime, the individual magnitude of flux for the three ions is similar, with P being the smallest in magnitude. In this slow diffusion regime, the ionic flux exhibits small fluctuations around the zero-flux axis, indicating that ions remain mobile but diffuse at a significantly slower rate. Overall, Li ions continue migrating toward the SSE, while S and P ions move toward the Li metal anode. All three flux curves gradually approach the zero-flux axis, suggesting that the 10 ns simulation nears interphase passivation.

**Figure 7:**
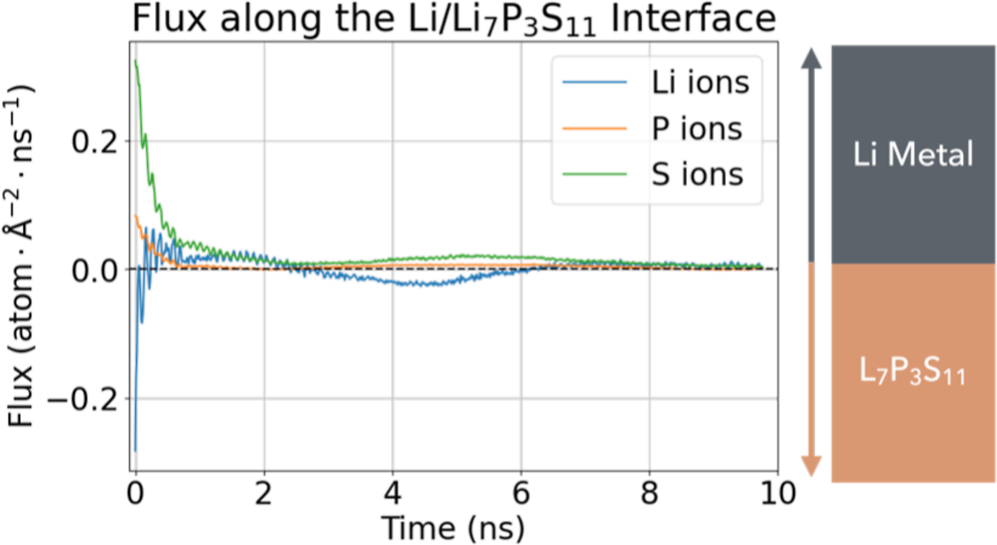
Net flux for Li-, P-, and S-ion species across the Li/Li_7_P_3_S_11_ interface throughout the 10 ns *NpT* simulation. Positive flux denotes ion migration toward the Li metal anode; negative flux denotes ion migration toward the Li_7_P_3_S_11_ SSE.

We performed multiple small-scale MD simulations with the final ACE MLIP model and obtained the Onsager transport coefficients for an amorphous configuration corresponding to each stable phase in the Li–P–S compositional space according to the Materials Project. The flux of Li, P, and S ions in the phases was calculated via eqs 1 and 2, as described in the Onsager Ionic Transport Analyses section and the Supporting Information. Figure 8 demonstrates three key takeaways: (1) the Li-ion flux across the relevant Li–P–S ternary phase space is consistently negative (from Figure 8a), indicating migration toward the SSE only; (2) in contrast, S ions exhibit consistently positive flux in these phases (see Figure 8b), signaling migration toward the Li metal anode; and notably, (3) P ions exhibit flux behavior that varies directionally depending on the phase composition (see Figure 8c). Specifically, P ions tend to move toward the anode in S-rich regions and toward the SSE in Li-rich regions. This dependence on the local environment necessarily originates from the cross-correlation transport coefficients. Figure S9 describes the mean absolute Onsager transport coefficients for P correlated with Li, P, and S in phases with the compositional system Li–P, Li–P–S, and Li–S at 900 K, calculated using the same Onsager coefficients derived from Figure 8, and shows the magnitude of the P–P cross-correlated motion to be consistently lower than Li–P-correlated transport and on par with P–S. In summary, the direction and magnitude of P-ion migration depend on the local environment, resulting in the potential trapping of Li_*x*_P phases within the SEI.

**Figure 8:**
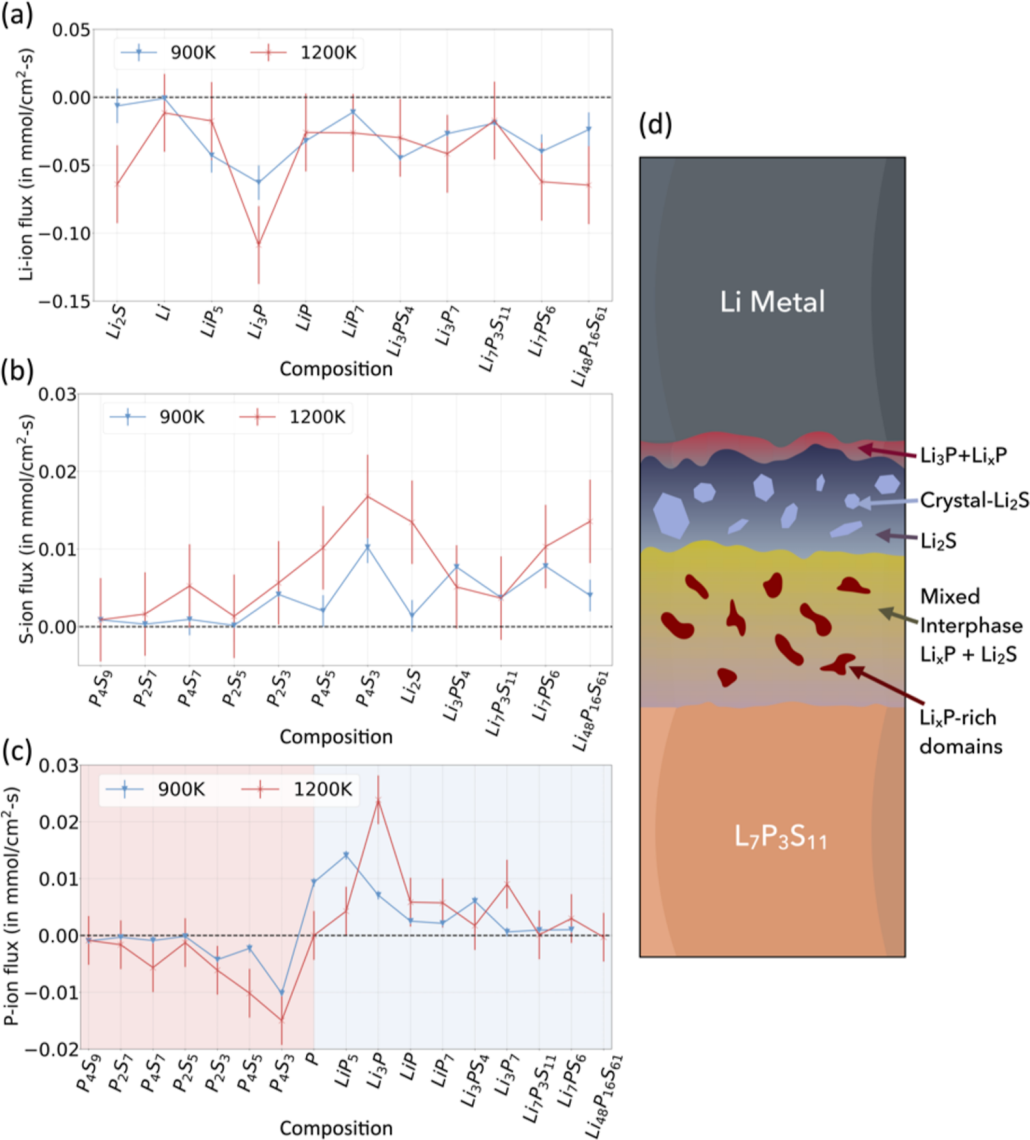
Correlated ionic flux calculated with respect to the host composition system from MD simulations with the final generated fitted MLIPs for (a) Li ions, (b) S ions, and (c) P ions at 900 and 1200 K. The positive direction is defined as movement toward the Li metal anode. (d) Schematic representation of the resulting Li/Li_7_P_3_S_11_ interface exhibiting a heterogeneous layered amorphous structure morphology. In particular, we note the formation of Li_*x*_P-rich phases within the Li_2_S matrix which may be the result of kinetic trapping due to the strong influence of the local environment on P-ion flux.

Prior in situ XPS studies from Wenzel et al.^18^ and Wood et al.^19^ indicate an early formation of Li_*x*_P and Li_2_S species within the interphase, with Li_2_S identified as the majority specie. As the reaction proceeds, Li_*x*_P species are observed to transform into the thermodynamically stable composition of Li_3_P. Wenzel et al.^18^ observed that Li_*x*_P forms first, followed by a sharp increase in Li_2_S, which becomes the dominant SEI phase. Over longer time scales, Li_3_P emerges as the main P species as the initial Li_*x*_P phases undergo further reduction. Wood et al.^19^ found that the interphase consists of Li_3_P, Li_2_S, and other reduced P species, forming a heterogeneous layer structure following the order of Li metal anode/Li_3_P/Li_2_S/SSE.

In general agreement with experimental results, we identified a heterogeneous, layered amorphous interphase structure, exhibiting pockets of nanocrystalline Li_2_S. Figure 9 plots the evolution of the average coordination number of select ion pairs along the interface over the 10 ns ML-MD simulation. As shown in Figure 9a, the highest average number of Li-ion coordinations to P occurs in a narrow region near the Li metal anode, with a peak coordination number of 9.65 observed at 1.9–2.1 nm. As a comparison, the coordination of Li to P in Na_3_As-structured Li_3_P is 11. A secondary peak in coordination is observed in a band around 1.5 nm, followed by a gradual decrease in the average Li–P coordination toward the Li_7_P_3_S_11_ SSE. Figure 9c showcases the conjugate coordination (i.e., how many P are bonded to Li) and further illustrates this thin, high-coordination Li–P region at 1.9–2.1 nm near the Li metal anode, with a lower Li–P coordination zone located between 0.8 and 1.7 nm. Similar P–Li coordination numbers were reported in modeling studies by Camacho-Forero and Balbuena^23^ and Ren et al.,^49^ supporting the identification of Li_3_P as the likely dominant phase close to the Li metal. Compositional analysis of Li–P clusters within the interphase layer (Figure S5) reveals an average composition of Li_*x* = 4.35_P, where the elevated Li content (*x* > 3) likely reflects the kinetically slow formation of Li_3_P. Among the stable Li–P binary compounds (Li_3_P, LiP, Li_3_P_7_, and LiP_7_), Li_3_P uniquely lacks P–P coordination. The partial RDF analysis of the Li_3_P–Li_*x*_P-rich layer (see Figure S8d) shows the lack of P–P coordination and Li–P RDF peak occurring at 2.49 Å which also supports the predominant formation of Li_3_P.

**Figure 9:**
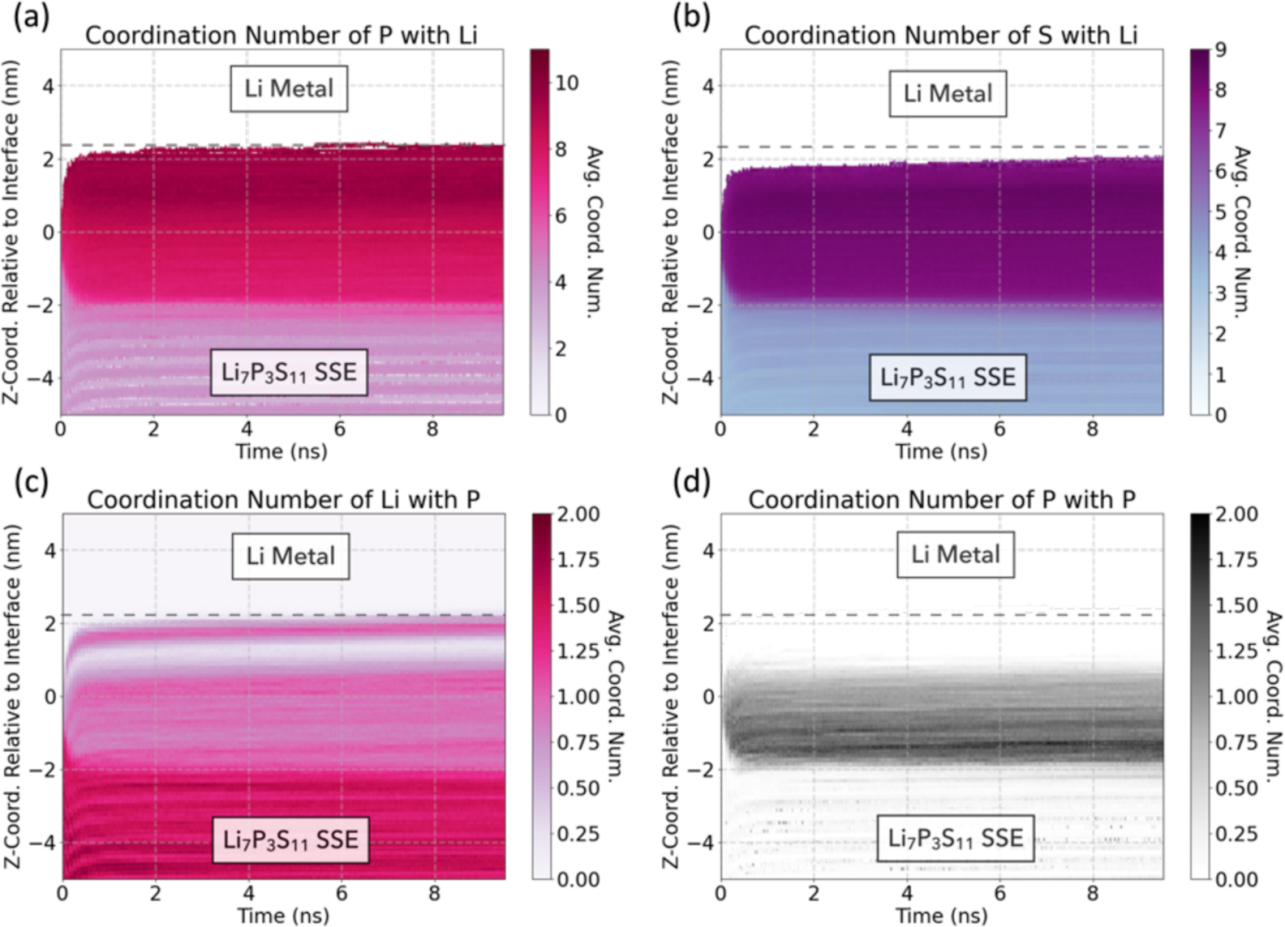
Spatial density distribution of averaged coordination numbers of (a) P sites with Li, (b) S sites with Li, (c) Li sites with P, and (d) P sites with P throughout the *NpT* 10 ns ML-MD simulation of Li/Li_7_P_3_S_11_. Note that the positive side of each plot depicts the Li metal region, and the negative side of each plot represents the Li_7_P_3_S_11_ SSE region. The dashed line indicates the approximate boundary between Li metal and the SEI. The *z*-coordinate is shifted relative to the center of the initial Li/Li_7_P_3_S_11_ interface. See Figures S6 and S7 for other species, full coordination numbers, and site presence.

Figure 9b displays the average S to Li coordination, which peaks at 8.25 in the SEI close to the Li metal anode, which should be compared to the coordination number of 8 Li to each S in antifluorite-structured Li_2_S. The two regions with the highest average S–Li coordination are separated by a layer of Li–P-rich phases.

The simulation also shows localized nanocrystalline Li_2_S domains starting to form between 1.7 and 2.0 nm from the Li metal (cf. Figure S3), at the beginning of the slow diffusion regime. Notably, this is the only crystalline phase observed in the SEI for the duration of the 10 ns simulation, with small nuclei forming within 1 ns and crystallizing into larger sites as the simulation proceeds. These nanocrystalline regions range from 3 to 6 nm radii, with a maximum detected size of 6.56 nm, and exhibit minimal P-ion defects (see Figure S3) with a well-defined periodic structure embedded in the amorphous Li_2_S matrix. Between the nanocrystalline domain of Li_2_S and the Li_7_P_3_S_11_ SSE domain, we observe a disordered, likely amorphous configuration of mixed Li_2_S and Li_*x*_P phases at various stoichiometries.

Figure 9d shows that regions in the SEI closest to the SSE show an increase in the P–P coordination number relative to the original Li_7_P_3_S_11_ coordination, suggesting the formation of P-rich Li–P phases such as LiP, Li_3_P_7_, and LiP_7_. The lack of P–S coordination in Figure S7f,h shows that polyanion groups, such as P_2_S_7_ and PS_4_, are not present in the SEI but remain in the Li_7_P_3_S_11_ layer. At the boundary between the interphase and the Li_7_P_3_S_11_ SSE, the SSE exhibits increased disorder, losing its partial crystallinity and periodicity. In summary, we find that the SEI forming between the Li metal anode and Li_7_P_3_S_11_ adopts a heterogeneous amorphous layered configuration of Li/Li_3_P–Li_*x*_P/Li_2_S/mixed-Li_2_S–Li_*x*_P/disordered-Li_7_P_3_S_11_/Li_7_P_3_S_11_, as shown in Figure 8d.

Finally, we comment on the rate of reaction, which is undeniably faster than anticipated from room temperature experiments. The interface develops passivation within 10 ns, which is significantly faster than observations by Wenzel et al.,^18^ where the deposition of Li on the Li_7_P_3_S_11_ powder found that the relative molar fraction of the phases in the SEI still fluctuates after 1 h. Our simulation also does not capture the SEI morphology after cycling of the SSB system. We suggest that one reason for the increased rate of reactivity is the well-known tendency for MLIPs to overestimate diffusion and ionic mobility, as discovered by Zheng et al. when attempting to reproduce diffusivity data from amorphous AIMD calculations.^72^ It is also possible that the atom-perfect interface matching in the simulations increases the rate of reaction.

We also caution that the MLIP will not overcome any of the inherent limitations of its training data, e.g., the DFT level of theory. For example, the functional of choice here: PBE, does not account for long-range dispersion interactions, and they are therefore missing in the MLIP. An explicit dispersion correction could be incorporated into the model afterward or adjusted in training. Additionally, simpler functionals like PBE struggle to describe stretched radical bonds^90^ and tend to predict incorrect charge transfer between reactant and product atoms during bond breaking and formation. Both effects are exacerbated in small subcell representations of the partly reacted interface, which may not be charge balanced. While higher-level approximations in DFT or wave function methods alleviate both issues, they are currently inaccessible in solid-state calculations at a high-throughput scale. Other effects like periodic boundary condition errors shown by Zhong et al.^91,92^ demonstrate issues that plague energy and force accuracy for amorphous structures.

## 4 Conclusion

This study presents a framework that integrates a DFT-based data generation scheme with an active learning loop to efficiently sample the potential energy surface for training customized machine-learning interatomic potentials (MLIPs) using the Atomic Cluster Expansion (ACE) method. We demonstrate this approach by modeling the interphase reaction between a Li metal anode and solid-state electrolyte Li_7_P_3_S_11_. We observe atomistic passivation of the final heterogeneous layered interphase within the 10 ns time frame of the *NpT* simulation conducted at 300 K, which indicates that the MLIP overestimates ionic diffusion. Nevertheless, our MLIP model exhibits strong predictive capabilities in terms of the chemical and morphological character of the resulting SEI. It successfully identifies the early formation of Li_*x*_P, followed by Li_2_S, which are thermodynamically stable under ambient conditions and have been observed experimentally.^18,19^ As the reaction progresses, Li_*x*_P preferentially transforms into Li_3_P. The interphase reactivity exhibits two distinct kinetic regimes: an initial fast diffusion regime that occurs within 20 ps, followed by a slow diffusion regime characterized by the emergence of a heterogeneous layered structure with the sequence of phases including Li, Li_3_P–Li_*x*_P, Li_2_S, mixed Li_2_S–Li_*x*_P, disordered Li_7_P_3_S_11_, and Li_7_P_3_S_11_. Our simulations demonstrate that the formation of the heterogeneous layered interphase is primarily driven by the rapid formation of Li_*x*_P, which is subsequently followed by the formation of Li_2_S. Using Onsager analyses of cross-correlated ionic mobility across amorphous phases of varying interfacial compositions across the interface, we find that Li and S ions exhibit consistent directional flux, irrespective of the composition. In contrast, the flux of P ions exhibits a strong dependence on its local chemical environment, which enables the kinetic trapping of Li_*x*_P phases within the SEI. Li_2_S is observed to initially form an amorphous layer, from which nanocrystalline domains begin to form from the amorphous matrix. Future research will focus on extending the simulation time scale to explore other kinetic phenomena.

## Supplementary Material



## Data Availability

To support reproducibility, the datasets and models featured in this work can be obtained from the corresponding author upon request.

## Notes

VASP input settings used for DFT calculations; architecture of the final fitted ACE potential, validation plots of the potential, EOS table information, and additional RDF plots; and supplementary figures and plots on the large-scale MD simulations performed on Li/Li_7_P_3_S_11_ regarding site presence, coordination numbers, snapshots of simulations to qualitatively define Li_2_S nanocrystalline domains, and additional flux data calculated from Onsager transport coefficients (PDF)
