# Supplementary material for: An Atomistic Study of Reactivity in Solid-State Electrolyte Interphase Formation for Li/Li7P3S11
Source: J Phys Chem C Nanomater Interfaces. 2025 Sep 3;129(36):16043–54. doi: 10.1021/acs.jpcc.5c03589 (PMC12434724; doi:10.1021/acs.jpcc.5c03589)
Supplement: Supplementary file 1 [file jp5c03589_si_001.pdf]

Supporting Information: An Atomistic Study of Reactivity in Solid State Electrolyte Interphase Formation for $Li/Li_7P_3S_{11}$ Systems

Bryant Y. Li,[*,†] Vir Karan,[†] Aaron D. Kaplan,[‡] Mingjian Wen,[‡] and Kristin A. Persson[*,†,‡]

†*Department of Materials Science and Engineering, University of California Berkeley, Berkeley, California 94720, United States*

‡*Materials Science Division, Lawrence Berkeley National Laboratory, Berkeley, California 94720, United States*

E-mail: bryant_li@berkeley.edu; kapersson@lbl.gov

ACE Potential Architecture

The Atomic Cluster Expansion (ACE) model proposed by Drautz is integrated into the `pacemaker` Python package.[S2–S4] ACE fits an interatomic potential based on the energies and forces present in the structural configuration, and is able to perform classical MD simulation with said trained potential. The ACE potential parametrizes local atomic environments using a basis set comprised of spherical harmonics, truncated at a maximum angular momentum of $\ell_{\max}$, in conjunction with radial basis functions represented by the first $n_{\max}$ power-law scaled Chebyshev polynomials.[S3,S5] Atomic interactions are limited to a selected cutoff radius, at which point radial components approach zero. The atomic basis functions are further reduced to a set of symmetrically invariant basis functions, denoted as the $\mathbf{B}$ matrix. The coefficients, $c_{n\ell}^{(K)}$, serve as trainable parameters for fitting the model, and are optimized to minimize the loss function. The total energy of the system is expressed as:

$$E_i(\sigma, \mu) = \sum_{Kn\ell} c_{n\ell}^{(K)} \mathbf{B}_{n\ell} \tag{1}$$

where i represents the atomic index, σ denotes the collection of vectors between atom i and its neighbors $(r_{1i}, r_{2i}, ..., r_{Ni})$, and μ represents the associated list of chemical species of each atom $(\mu_{1i}, \mu_{2i}, ..., \mu_{Ni})$. The multibody order of the interaction, K, is defined as the number of atoms involved in the interaction (e.g., a four-body interaction corresponds to $K = 4$). The optimization of the coefficients, $c_{n\ell}^{(K)}$, is performed by minimizing the loss function, Λ, which is given by:

$$\Lambda = (1 - \kappa)\Delta_E^2 + \kappa\Delta_F^2 \tag{2}$$

The weighting coefficient κ determines the relative contribution of the squared deviation in predicted energies, Δ_E^2, and squared deviation in predicted forces, Δ_F^2, to the total loss function, Λ.

For a more detailed discussion of the Atomic Cluster Expansion (ACE) framework, we refer the reader to the work of Drautz.[S1] The ACE model was selected for it's scalability,

intepretability, and efficient $O(n)$ scaling with MPI parallelization. Preliminary tests shows that DeepMD-kit,[S6] Allegro,[S7] NequIP,[S8] and fine tuning universial interatomic potentials such as M3GNet,[S9] CHGNet,[S10] MACE[S11] are also possible, and could be investigated in future work.

Li-P-S ACE Potential

Table S1: Final ACE potential architecture.

Elements	max-body order	n_{max}	ℓ_{max}
Uniary	five-body	15/3/2/1	0/4/2/0
Binary	four-body	15/3/2/1	0/4/2/0
Ternary	five-body	15/3/2/1	0/4/2/0

VASP input settings

We provide an overview of the VASP input settings used in this work. While we recommend using the `StaticMaker` and `RelaxMaker` VASP classes in `atomate2`, Tables S2 and S3 provide detailed input settings used for the INCAR and POTCAR (pseudpotentials) respectively. Note that the k-point density was set to 64 points/Å^{-3}, and 200 in metals. The KPOINTS files are made by the `Kpoints.automatic_density` function in `pymatgen`.

Table S2: VASP INCAR parameters used for the structural relaxations in this work.

INCAR tag	Value
ALGO	Fast
EDIFF	10^{-5}
EDIFFG	-0.02
ENAUG	1360
ENCUT	680
GGA	PS
IBRION	2
ISIF	3
ISMEAR	0
ISPIN	2
LAECHG	True
LASPH	True
LCHARG	True
LDAU	True
LDAUPRINT	1
LDAUTYPE	2
LELF	False
LMIXTAU	True
LORBIT	11
LREAL	False
LVTOT	True
LWAVE	False
NELM	200
NSW	99
PREC	Accurate
SIGMA	0.05

Table S3: VASP pseudopotentials (`POTCAR`) used for this work. The second column indicates the "TITEL" keyword of the POTCAR, and the third column indicates a derived SHA1 hash from the POTCAR text. To compute SHA1 values, we first replace all space characters with empty strings, and then call python's `hashlib.sha1` function on the space-removed string, followed by `hexdigest`. This work used the "PBE 54" PAW pseudopotential library. The precise input settings are defined in the `_BASE_VASP_SET` variable of `atomate2.vasp.sets.base`.

Element	POTCAR TITEL	SHA1
Li	PAW_PBE Li_sv 10Sep2004	dac9b677547cd436de3ebde1eaefbc5d99158956
P	PAW_PBE P 06Sep2000	e8d4fb0be7ea32a04e2d8821a4d8fc58bc0105b0
S	PAW_PBE S 06Sep2000	682dce0979a7aefa95a46a9159e96cef62516342

Validation Metric Plots

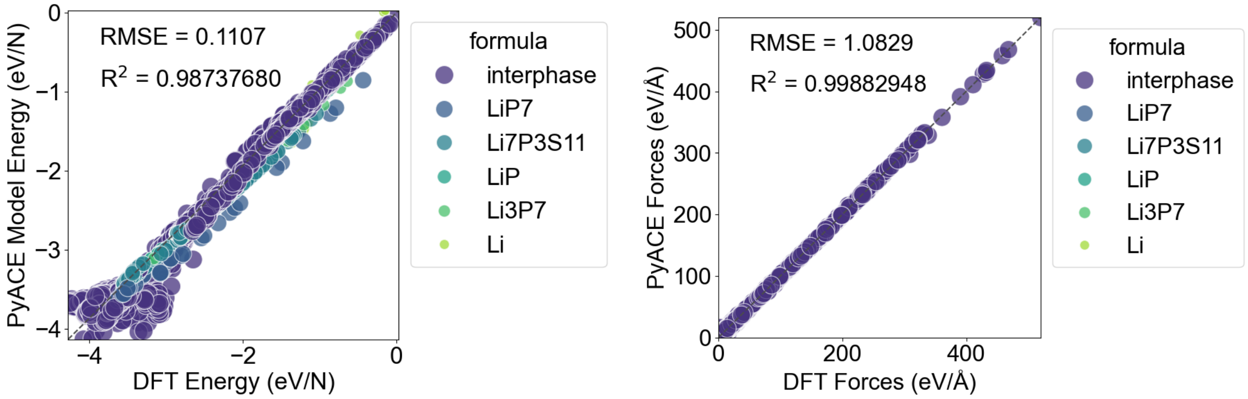

(a) (b)

Figure S1: Final generation of fitted PyACE (pacemaker) ML-IAP model benchmarked against DFT data for (a) energy and (b) forces

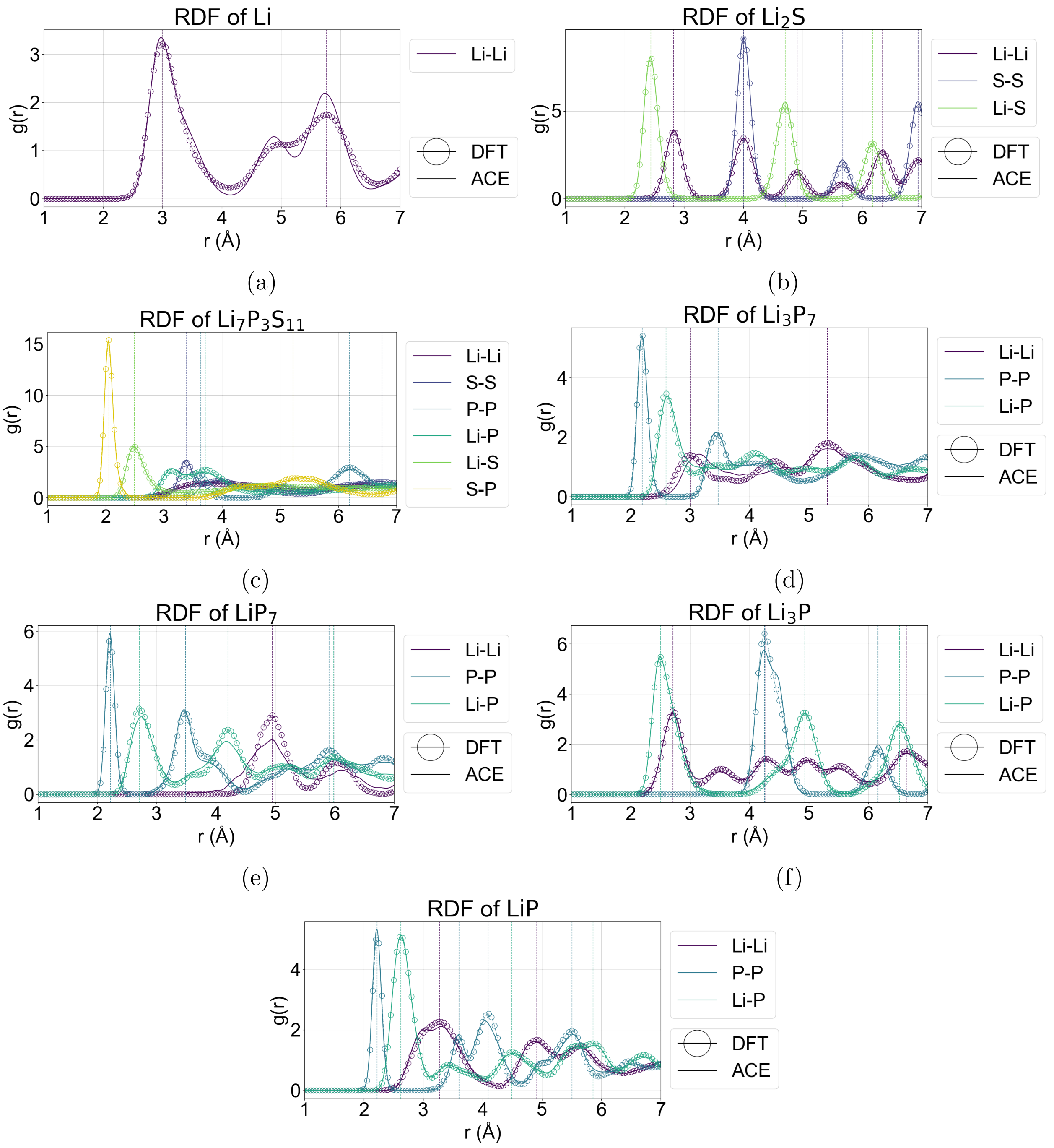

Figure S2: Radial distribution functions for all the benchmark phases

Table S4: RDF peak locations (Å) for all specie pairs in all benchmark phases from the AIMD data, calculated via scipy find peaks function

**Specie Pair**	**Li**	**Li_2S**	**Li_3P**	**Li_3P_7**	**LiP_7**	**LiP**	**$Li_7P_3S_{11}$**
Li–Li	2.99, 5.76, 7.56	2.82, 4.0, 4.9	2.71, 4.27, 6.64	3.0, 5.31, 7.75	4.95, 6.0, 7.62	3.27, 4.91, 7.84	/
Li–P	/	/	2.5, 4.93, 6.52	2.59	2.71, 4.2, 5.98	2.62, 4.49, 5.86	3.71
Li–S	/	2.44, 4.7, 6.17	/	/	/	/	2.49
P–P	/	/	4.25, 6.16, 7.42	2.19, 3.47	2.22, 3.48, 5.9	2.22, 3.6, 4.09	3.63, 6.19
P–S	/	/	/	/	/	/	2.05, 5.22
S–S	/	4.0, 5.67, 6.94	/	/	/	/	3.39, 6.75

Table S5: Comparison of ACE and DFT calculations for compounds in the dataset

Compound	ACE				DFT			
	E_0 (eV)	B_0 (GPa)	B_1	V_0 (Å^3)	E_0 (eV)	B_0 (GPa)	B_1	V_0 (Å^3)
$Li_{12}P_{28}$	-1.40×10^2	7.97	3.9448	7.91×10^2	-1.89×10^2	8.16	5.3035	8.41×10^2
$Li_{14}P_6S_{22}$	-1.56×10^2	7.67	4.8248	8.76×10^2	-1.85×10^2	6.78	6.3864	9.18×10^2
Li_{16}	-2.66×10^1	7.25	3.6841	3.36×10^2	-3.05×10^1	8.98	3.4874	3.23×10^2
$Li_{16}P_{112}$	-4.83×10^2	7.54	3.8252	2.72×10^3	-6.57×10^2	9.14	4.5481	2.94×10^3
$Li_{48}P_{16}$	-1.80×10^2	2.418	3.4071	9.47×10^2	-2.24×10^2	2.606	3.8773	9.33×10^2
Li_8P_8	-5.25×10^1	1.954	3.5642	2.61×10^2	-6.69×10^1	1.942	5.2757	2.56×10^2
Li_8S_4	-4.25×10^1	2.612	4.0941	1.79×10^2	-4.80×10^1	2.671	4.0217	1.85×10^2

Calculating Flux from Onsager Transport Coefficients

From the chemical potential diagram[S12] and previously shown by Karan et. al[S13] we calculate the chemical potential gradients:

$$\nabla\tilde{\mu}_{900\text{K}} = \begin{bmatrix} \nabla\tilde{\mu}_{\text{Li}} \\ \nabla\tilde{\mu}_{\text{P}} \\ \nabla\tilde{\mu}_{\text{S}} \end{bmatrix} = \begin{bmatrix} 1.62199365 \\ -1.65941488 \\ -3.24398729 \end{bmatrix}$$

$$\nabla\tilde{\mu}_{1200\text{K}} = \begin{bmatrix} \nabla\tilde{\mu}_{\text{Li}} \\ \nabla\tilde{\mu}_{\text{P}} \\ \nabla\tilde{\mu}_{\text{S}} \end{bmatrix} = \begin{bmatrix} 1.43008018 \\ -1.48392807 \\ -2.86016035 \end{bmatrix}$$

These $\nabla\tilde{\mu}$ are used in Eq.2 together with the Onsager transport coefficients to obtain the ionic flux. It should be noted that the Onsager transport coefficients consists of $L_{\text{Li-Li}}$, $L_{\text{Li-P}}$, $L_{\text{Li-S}}$, $L_{\text{P-Li}}$, $L_{\text{P-P}}$, $L_{\text{P-S}}$ for the Li-P-S system, and these coefficients are fitted for each MD trajectory of a specific amorphous composition. As an example, performing a ML-MD simulation on amorphous $Li_7P_3S_{11}$ at 900 K would result in one set of Onsager transport coefficients. These coefficients and $\nabla\tilde{\mu}_{900\text{K}}$ for each respective ion (Li, P, and S) are then used to calculate the respective ionic flux at the Li/$Li_7P_3S_{11}$ interface (see Fig. 9(a),(b),(c) and Supplementary Fig. S10.)

Cluster Analyses of Li_2S and Li_3P rich SEI Layers

Fig. S7 shows that the average coordination numbers (CNs) for S–Li and P–Li are 8.25 and 9.65, respectively, consistent with reference data from the Materials Project: S is coordinated to 8 Li in Li_2S, and P to 11 Li in Li_3P. CN analysis of Li–S further supports the presence of Li_2S, with our data showing an average CN of 3.49 for Li around S in the proposed Li_2S layer — close to the crystalline value of 4. The slightly lower coordination is expected for amorphous materials, as described by Zachariasen's rules.[S14]

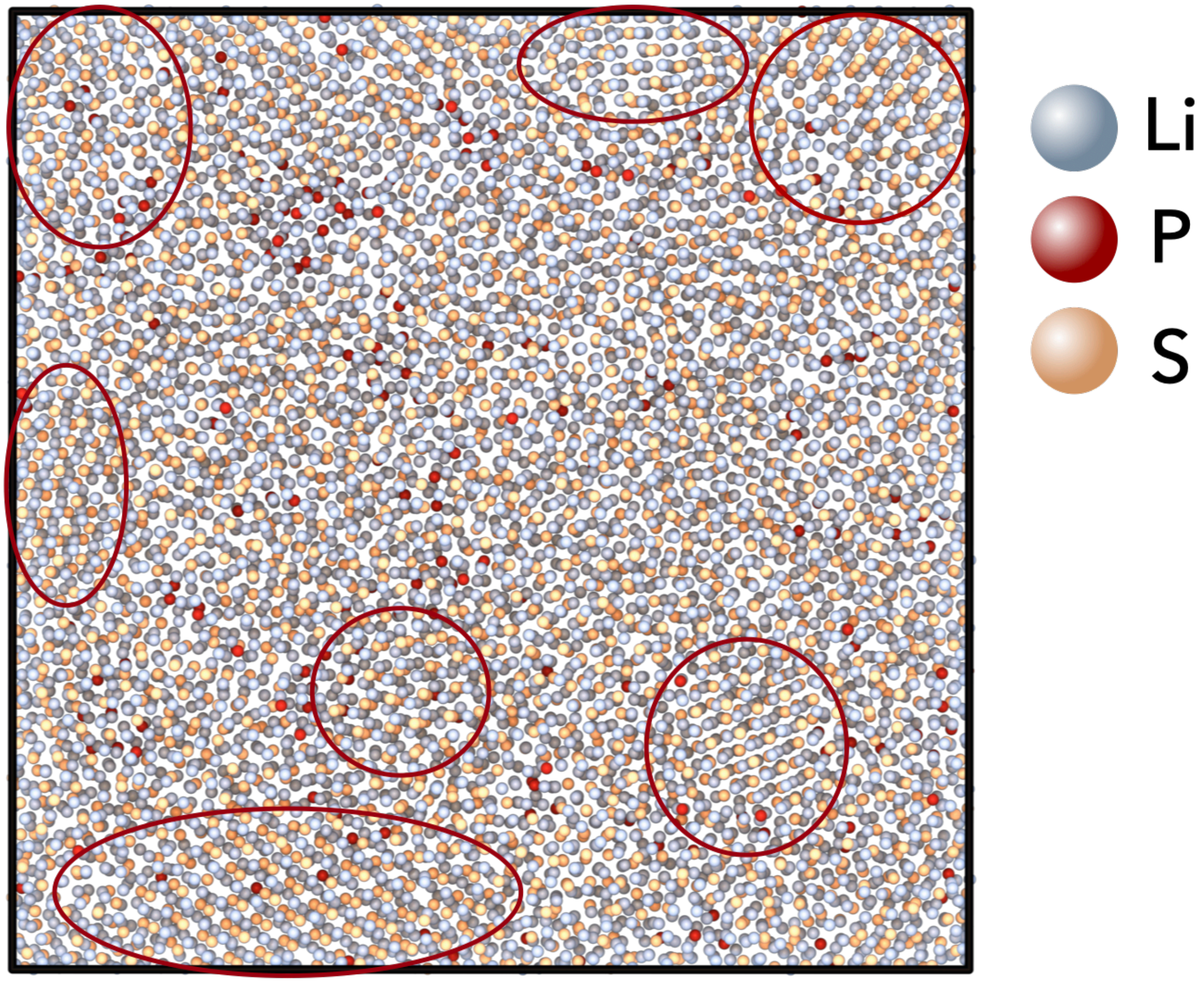

Figure S3: Final snapshot from a 10ns NpT simulation of (100)/(100) Li/$Li_7P_3S_{11}$ interface viewed along the c-axis (top-down perspective), showing the interfacial Li_2S domain layer only. Maroon circles denote regions of nanocrystalline Li_2S formation within the Li_2S domain.

Fig. S4 visually illustrates the presence of Li-only domains and the spatial inhomogeneity of detectable Li_xP clusters in the Li-P rich layer around 1 nm from the Li metal boundary or 1.9–2.1 nm from the initial interface. To obtain statistically sound estimates of the local stoichiometry in the Li-P clusters, we conducted a cluster analysis on the Li_xP-rich SEI layers, and benchmarked it against the better evidenced Li_2S-rich layer. Using the Ovito[S15] cluster analysis functionality and a cutoff distance of 2.7Å, chosen to include longest bond length observed for both Li-P and Li-S bonds (see Fig. S2), we determine the 3 largest clusters observed in the layers. The results are shown in the violin plot Fig. S5 which depicts the stoichiometric ratio distribution between Li-S and Li-P in their respective layers (1.7–2.0 nm and 1.9–2.1 nm) observed for the final 5 ns of the NpT MD Li/$Li_7P_3S_{11}$ interface simulation. The mean stoichiometric ratios are $Li_{2.38}S$ and $Li_{4.35}P$, respectively with a much larger spread of compositions for the P-rich domain.

Fig. S8d shows the partial radial distribution function for the same Li-P-rich layer, close to the Li metal anode. The first peak for the Li-P curves quantitatively matches with the Li_3P peak in Table. S4, which corresponds to at 2.49Å. Further, the lack of P-P bonding in Fig. S8d supports the absence of more P-rich Li-P binaries such as LiP, Li_3P_7, and LiP_7.

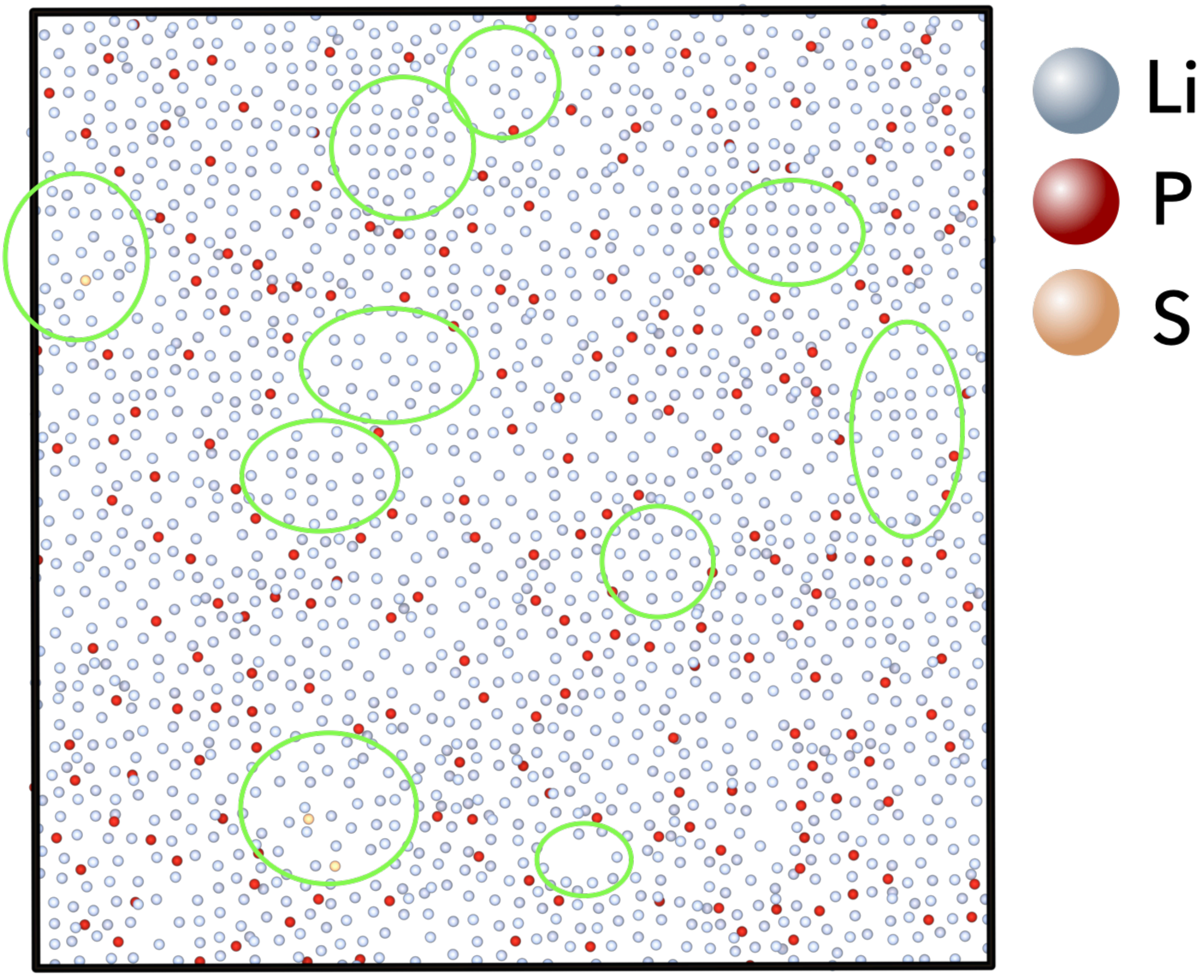

Figure S4: Final snapshot from a 10ns NpT simulation of (100)/(100) Li/$Li_7P_3S_{11}$ interface viewed along the c-axis (top-down perspective), showing the interfacial proposed Li_3P domain layer only. Lime circles denote regions of Li only clusters.

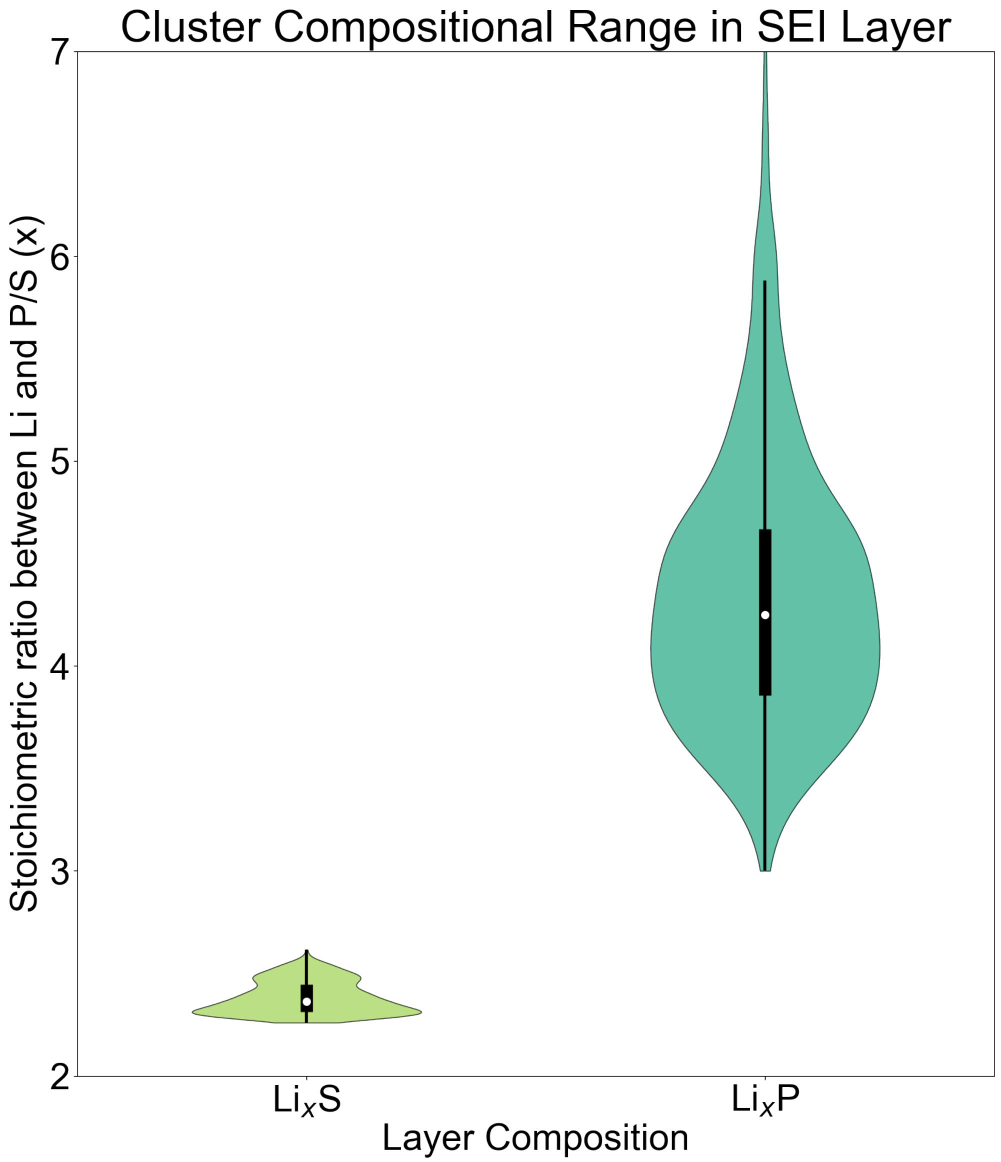

Figure S5: Cluster compositional analysis of SEI's Li_2S and Li_3P layers in the main NpT 10 ns MD simulations. Violin plot demonstrates the spread of compositional ranges observed from the largest cluster at each timeframe at the given layer. The plot captures the the cluster stoichiometric ratios observed in each of the layers.

Supplementary figures and plots on MD simulations

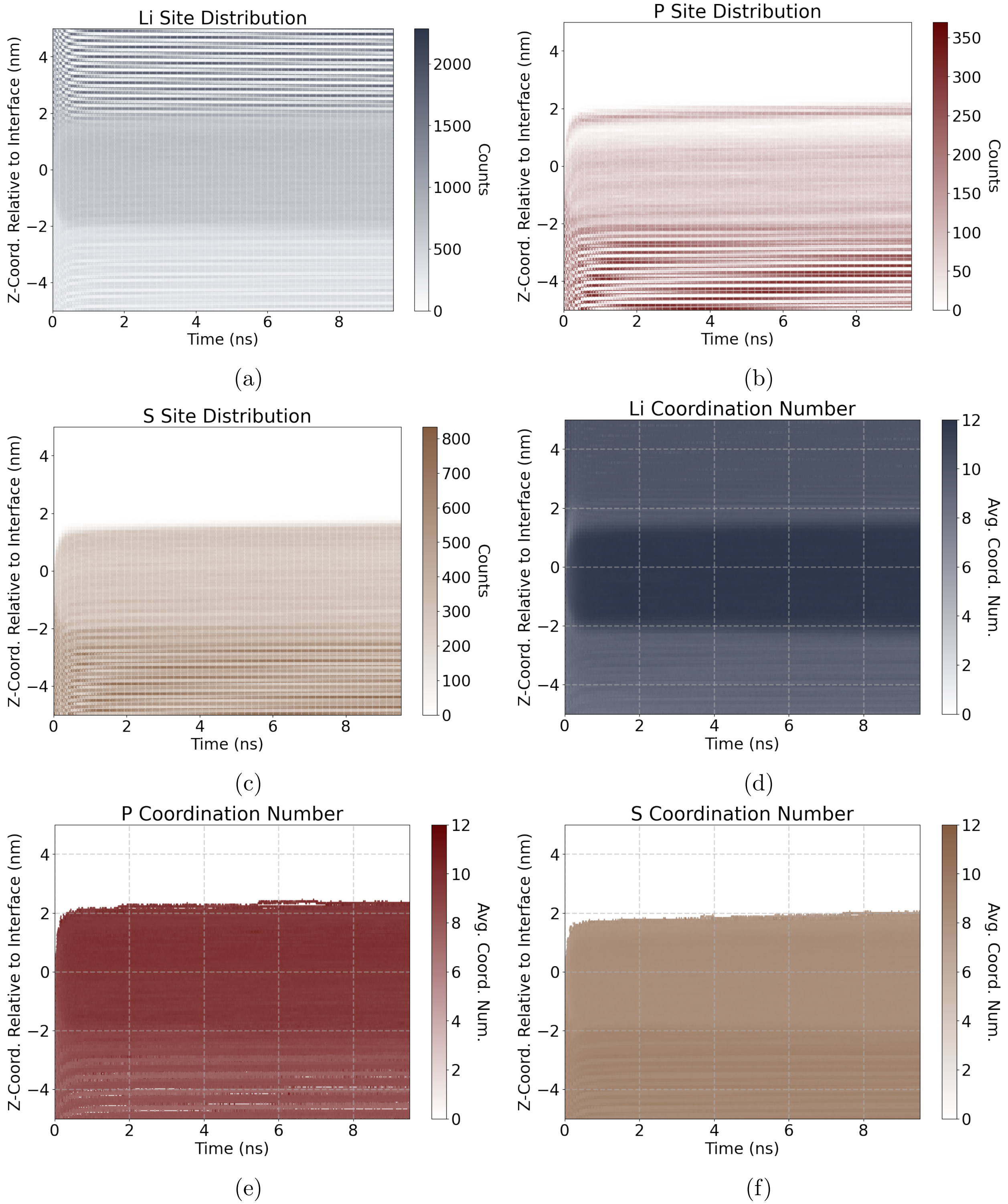

Figure S6: Atomic species distributed across the interfaces and their respective coordination numbers as a function of space and time.

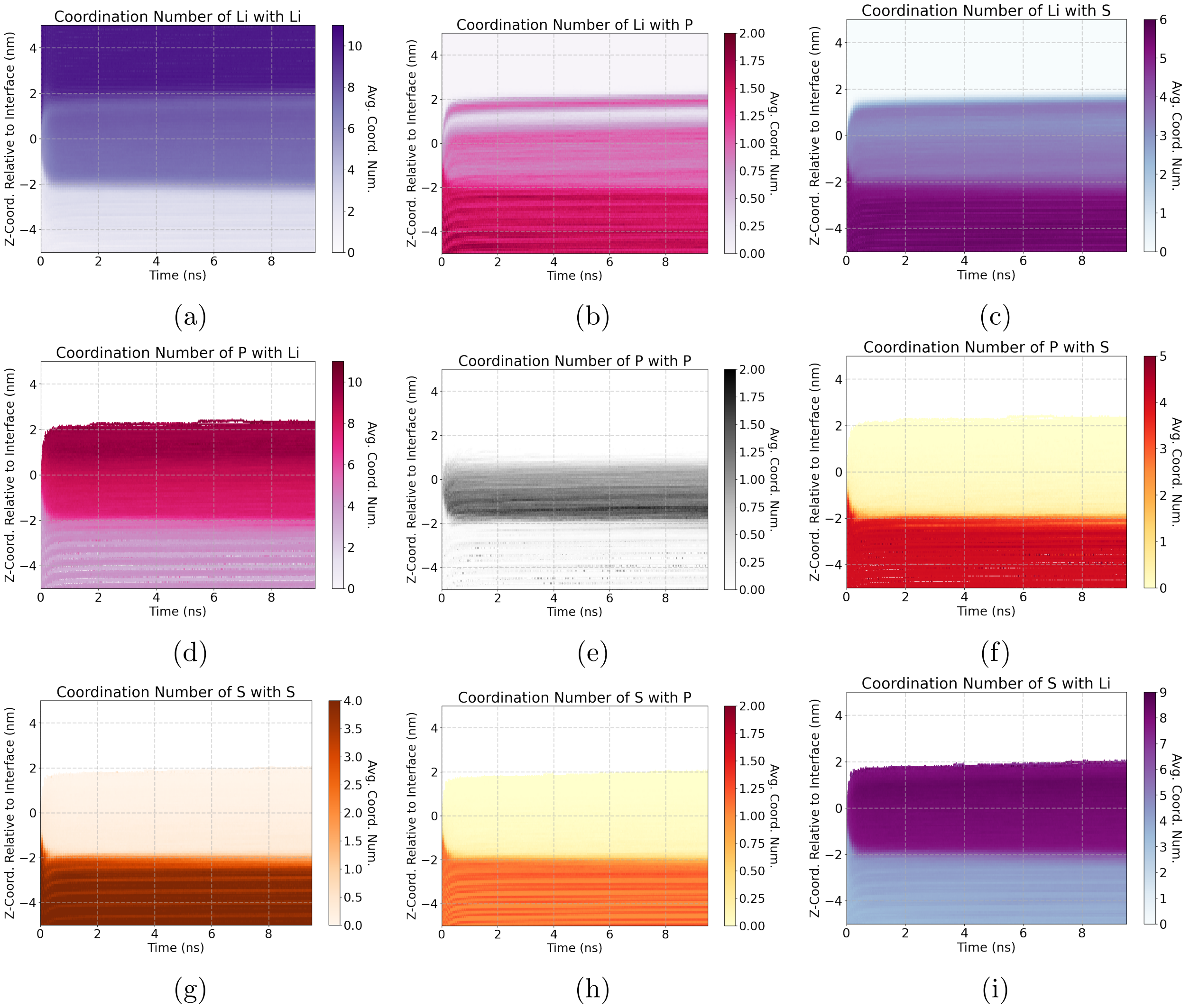

Figure S7: Pairwise coordination number of atomic species distributed across the interface over the simulated reaction

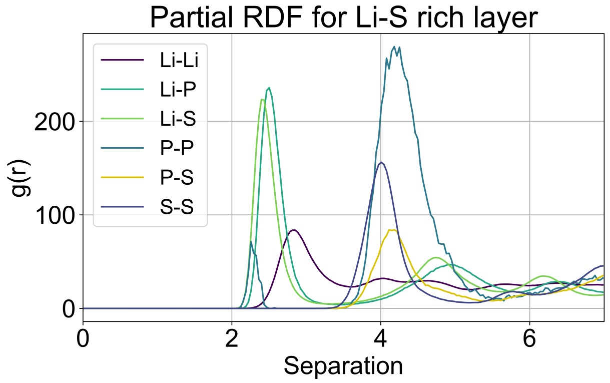

(a) All pairwise species, located at relative z-height of 1.7–2.0 nm

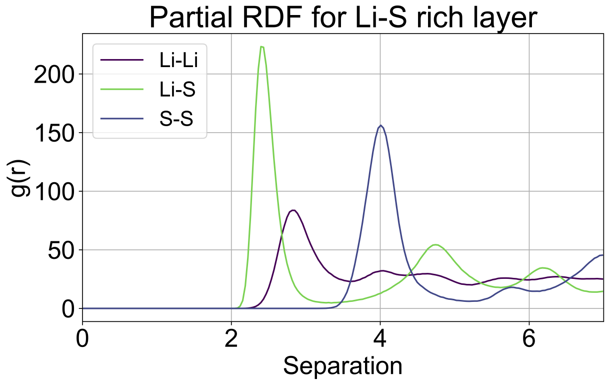

(b) Li-S interactions only, located at relative z-height of 1.7–2.0 nm

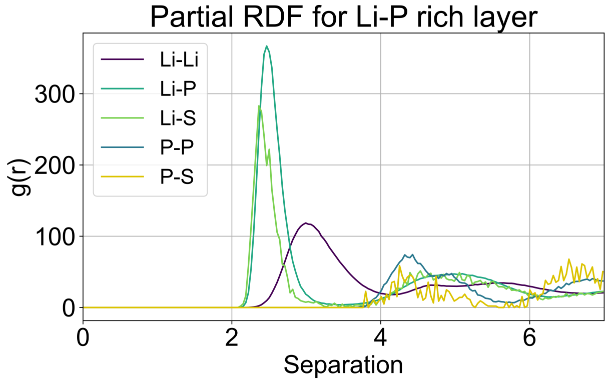

(c) All pairwise species, located at relative z-height of 1.9–2.1 nm

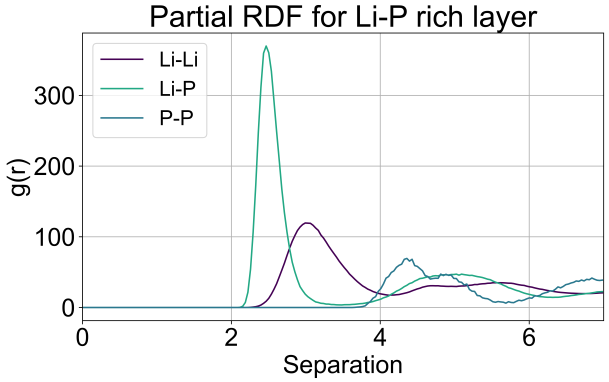

(d) Li-P interactions only, located at relative z-height of 1.9–2.1 nm

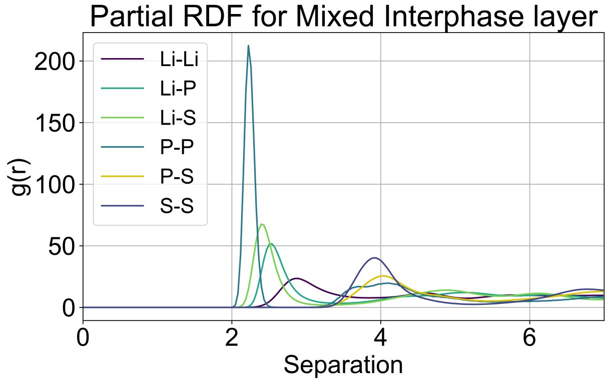

(e) All interactions centered around the center of the mixed SEI layer, located at relative z-height of -2.1-1.5 nm

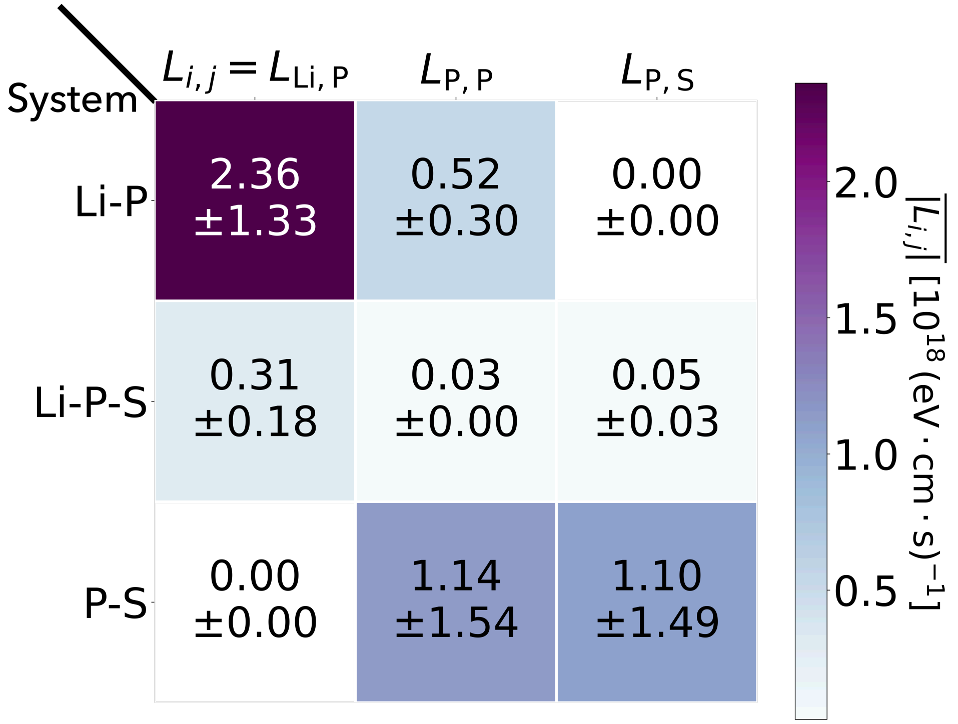

Figure S9: Mean absolute Onsager transport coefficients for P correlated with Li, P, and S in amorphous phases with the compositional ranges of Li-P, Li-P-S, and Li-S at 900 K, with standard deviation. The mean absolute Onsager coefficients were calculated from the Onsager matrix shown in Fig. 8. The magnitude of the of the coefficients corresponds with the dominant transport coefficient driving the P ions.

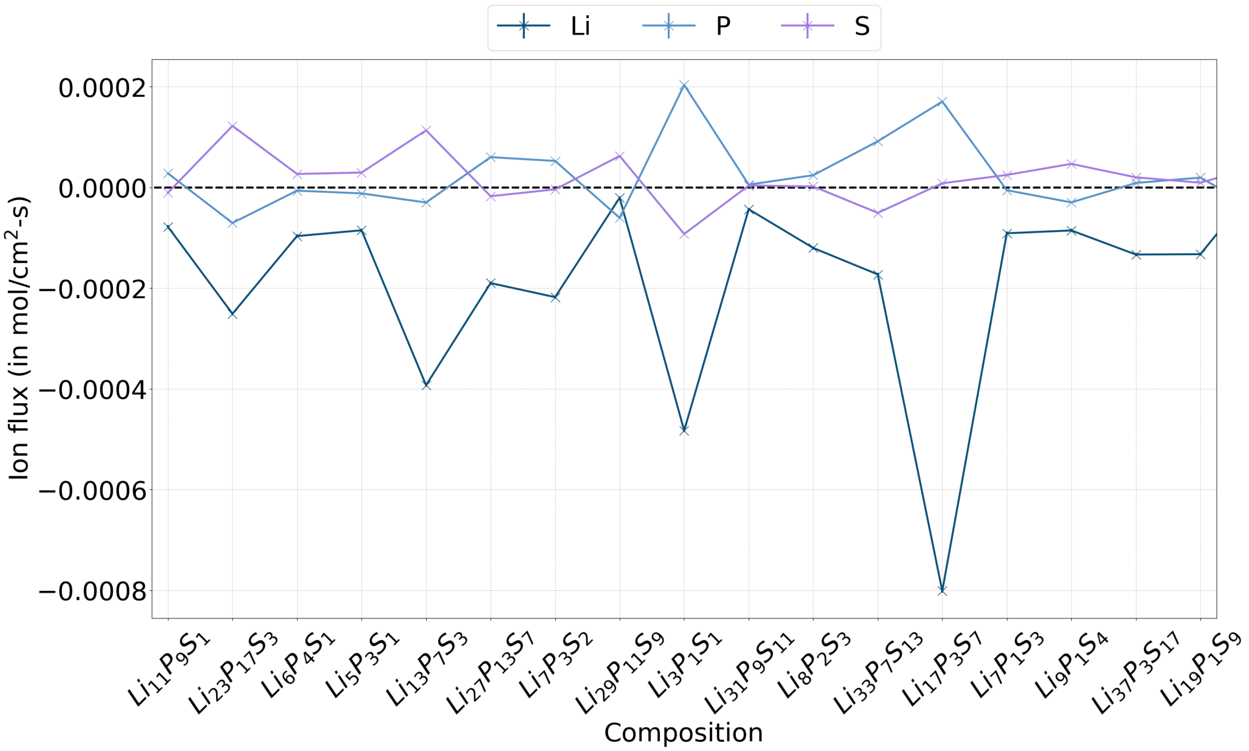

Figure S10: Ionic flux for Li, P and S in amorphous phases corresponding to the compositions on the x-axis, covering the range from LiP to Li_2S. These compositional ranges represent the local compositions found in the mixed LiP/Li_2S amorphous interphase layer. The figure highlights the low magnitude of fluxes of P and S ions. Li ions exhibit a strong flux towards $Li_7P_3S_{11}$.

References

(S1) Drautz, R. Atomic cluster expansion for accurate and transferable interatomic potentials. *Physical Review B* **2019**, *99*, 014104.

(S2) Lysogorskiy, Y.; Bochkarev, A.; Mrovec, M.; Drautz, R. Active learning strategies for atomic cluster expansion models. *Physical Review Materials* **2023**, *7*, 043801.

(S3) Bochkarev, A.; Lysogorskiy, Y.; Menon, S.; Qamar, M.; Mrovec, M.; Drautz, R. Efficient parametrization of the atomic cluster expansion. *Physical Review Materials* **2022**, *6*, 013804.

(S4) Lysogorskiy, Y.; Oord, C. V. D.; Bochkarev, A.; Menon, S.; Rinaldi, M.; Hammerschmidt, T.; Mrovec, M.; Thompson, A.; Csányi, G.; Ortner, C.; Drautz, R. Performant implementation of the atomic cluster expansion (PACE) and application to copper and silicon. *npj Computational Materials* **2021**, *7*, 97.

(S5) Dusson, G.; Bachmayr, M.; Csányi, G.; Drautz, R.; Etter, S.; Van Der Oord, C.; Ortner, C. Atomic cluster expansion: Completeness, efficiency and stability. *Journal of Computational Physics* **2022**, *454*, 110946.

(S6) Wang, H.; Zhang, L.; Han, J.; E, W. DeePMD-kit: A deep learning package for many-body potential energy representation and molecular dynamics. *Computer Physics Communications* **2018**, *228*, 178–184, arXiv: 1712.03641.

(S7) Musaelian, A.; Batzner, S.; Johansson, A.; Sun, L.; Owen, C. J.; Kornbluth, M.; Kozinsky, B. Learning local equivariant representations for large-scale atomistic dynamics. *Nature Communications* **2023**, *14*, 579.

(S8) Batzner, S.; Musaelian, A.; Sun, L.; Geiger, M.; Mailoa, J. P.; Kornbluth, M.; Molinari, N.; Smidt, T. E.; Kozinsky, B. E(3)-equivariant graph neural networks for data-efficient and accurate interatomic potentials. *Nature Communications* **2022**, *13*, 2453.

(S9) Chen, C.; Ong, S. P. A universal graph deep learning interatomic potential for the periodic table. *Nature Computational Science* **2022**, *2*, 718–728.

(S10) Deng, B.; Zhong, P.; Jun, K.; Riebesell, J.; Han, K.; Bartel, C. J.; Ceder, G. CHGNet as a pretrained universal neural network potential for charge-informed atomistic modelling. *Nature Machine Intelligence* **2023**, *5*, 1031–1041.

(S11) Batatia, I.; Benner, P.; Chiang, Y.; Elena, A. M.; Kovács, D. P.; Riebesell, J.; Advincula, X. R.; Asta, M.; Avaylon, M.; Baldwin, W. J.; others A foundation model for atomistic materials chemistry. 2023; arXiv:2401.00096, 2023-12-29, 10.48550/arXiv.2401.00096 (accessed 2025-07-02).

(S12) Neilson, J. R.; McDermott, M. J.; Persson, K. A. Modernist materials synthesis: Finding thermodynamic shortcuts with hyperdimensional chemistry. *Journal of Materials Research* **2023**, *38*, 2885–2893.

(S13) Karan, V.; Gallant, M. C.; Fei, Y.; Ceder, G.; Persson, K. A. Ion correlations explain kinetic selectivity in diffusion-limited solid state synthesis reactions. 2025; arXiv:2501.08560, 2025-01-15, 10.48550/arXiv.2501.08560 (accessed 2025-07-02).

(S14) Mauro, J. C. *Materials kinetics: transport and rate phenomena*; Elsevier, 2020.

(S15) Stukowski, A. Visualization and analysis of atomistic simulation data with OVITO–the Open Visualization Tool. *Modelling and Simulation in Materials Science and Engineering* **2009**, *18*, 015012.
